# 3D Printed Mesh Geometry Modulates Immune Response and Interface Biology in Mouse and Sheep Model: Implications for Pelvic Floor Surgery

**DOI:** 10.1002/advs.202405004

**Published:** 2024-09-19

**Authors:** Kallyanashis Paul, Saeedeh Darzi, Cathal D. O'Connell, David M. Z. B. Hennes, Anna Rosamilia, Caroline E. Gargett, Jerome A Werkmeister, Shayanti Mukherjee

**Affiliations:** ^1^ The Ritchie Centre Hudson Institute of Medical Research Clayton 3168 Australia; ^2^ Department of Obstetrics and Gynaecology Monash University Clayton 3168 Australia; ^3^ Biofab3D@ACMD St Vincent's Hospital Melbourne VIC 3065 Australia; ^4^ Discipline of Electrical and Biomedical Engineering School of Engineering RMIT University Melbourne VIC 3000 Australia; ^5^ Pelvic Floor Disorders Unit Monash Health Clayton VIC 3168 Australia

**Keywords:** 3D‐printing, gynaecology, implant immune response, melt electrowriting, tissue engineering

## Abstract

Pelvic organ prolapse (POP) is a highly prevalent yet neglected health burden for women. Strengthening the pelvic floor with bioactive tissue‐engineered meshes is an emerging concept. This study investigates tissue regenerative design parameters, including degradability, porosity, and angulation, to develop alternative degradable melt electrowritten (MEW) constructs for surgical applications of POP. MEW constructs were fabricated in hierarchical geometries by two‐way stacking of the fibers with three different inter layer angles of 90°, 45°, or 22.5°. Implants printed at 22.5° have higher tensile strength under dry conditions and show better vaginal fibroblast (VF) attachment in vitro. In vivo assessment using preclinical mouse and ovine models demonstrates more effective degradation and improved tissue integration in 22.5° angular meshes compared to 90° and 45° meshes, with evidence of neo‐collagen deposition within implants at 6 weeks. The pattern and geometry of the layered MEW implants also influence the foreign body response, wherein the anti‐inflammatory phenotype shows a greater ratio of anti‐inflammatory CD206+ M2 macrophages/pro‐inflammatory CCR7+ M1 macrophages. This presents an attractive strategy for improving the design and fabrication of next‐generation vaginal implants for pelvic reconstructive surgery.

## Graphical abstract

Pelvic organ prolapse (POP) is a significant health challenge for women, especially with the lack of optimal surgical options post‐withdrawal of synthetic transvaginal meshes. The study pioneers degradable melt electrowrite vaginal implants with precise 22.5° angles and specific porosity. These configurations significantly influence macrophage behavior and gene expression, enhancing tensile strength, fibroblast attachment, and anti‐inflammatory response.

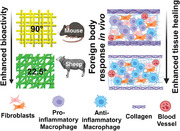

## 1 Introduction

Pelvic Organ Prolapse (POP) is a hidden yet debilitating urogynaecological disorder that negatively impacts the quality of life of 1 in 2 elderly women and 1 in 4 women across all age groups.^[^ ^1^ ^]^ POP primarily impacts elderly women without reliable treatment options with decreased quality of life, lost productivity, increased healthcare costs, and inequitable opportunities for women.^[^ ^2^ ^]^ The direct healthcare cost associated with POP exceeded USD $1 billion/year in the United States of America 25 years ago.^[^ ^3^ ^]^ A 100% growth in the aging population is predicted in the next 40 years, highlighting POP as a growing and unmet women's health need. POP is the herniation of pelvic organs, including the bladder, uterus, vault, and/or bowel, into the vagina and outside the body. Such a biomechanical failure primarily arises from stretch‐induced trauma of the maternal pelvic floor during vaginal birth, which injures the three levels of pelvic supports; the vaginal walls, the pelvic floor muscle complex, and the suspensory ligaments.^[^ ^4^, ^5^ ^]^ It is estimated that 60% of women experience vaginal trauma during parturition and severe birth injuries trigger the onset of chronic pelvic floor disorders like POP^[^ ^5^, ^6^ ^]^ in the later post‐menopausal stage of women's life. In addition, obstetric factors such as forceps delivery, prolonged second‐stage labour, considerable infant birth weight, anal sphincter injury, and episiotomy compound the risk of POP development in women.^[^ ^6^, ^7^, ^8^ ^]^ Forceps delivery^[^ ^6^ ^]^ adds about an extra 100,000 major cases per year of pelvic floor trauma.^[^ ^7^ ^]^ Thus, inadequate healing of birth injury has dire long‐term consequences on maternal pelvic floor health^[^ ^7^ ^]^ and yet, lacks a safe and reliable treatment. Patient factors compounding POP risk include overall parity, age, body mass index (BMI), chronic constipation, and coughing.^[^ ^9^ ^]^ Management options include conservative approaches such as pelvic floor physiotherapy and vaginal pessaries. However, surgical intervention is necessary for up to 19% of the female population.^[^ ^10^ ^]^ Until recently, non‐degradable polypropylene (PP) based transvaginal mesh kits were often used for reconstructive surgery to mitigate native tissue repair failures and reduce the risk of anatomical recurrence of POP. Despite strengthening the apical uterus leading to superior clinical and anatomical outcomes in reducing apical prolapse,^[^ ^11^, ^12^ ^]^ the transvaginal anterior and posterior vaginal wall repair with PP meshes has resulted in unacceptable complications such as mesh erosion, exposure, pain, and poor tissue integration^[^ ^13^, ^14^, ^15^ ^]^ that largely outweighed their benefits.^[^ ^16^ ^]^ In effect, transvaginal PP meshes were banned by regulatory bodies; TGA, MEDSAFE, FDA, and MHRA in Australia, NZ, USA, and UK, respectively. Due to the lack of innovative solutions in biomaterial design, surgeons have reverted to native tissue and biologic graft techniques, which have proven inadequate with high failure rates.^[^ ^17^, ^18^ ^]^ Reports from regulatory bodies have called for a new generation of biomaterial vaginal implants for improved surgical safety and efficacy in pelvic floor reconstructive surgery.^[^ ^19^, ^20^, ^21^ ^]^

The leading causal factor for which the non‐degradable transvaginal PP meshes were recalled is prolonged inflammation, resulting in an undesirable foreign body response (FBR) leading to poor tissue integration.^[^ ^15^, ^22^ ^]^ Prolonged FBR with over‐activation of enzymes such as matrix metalloproteinase‐2 results in disruption of vaginal smooth muscle and collagen loss and ultimately increased risk of erosions, exposure, and pelvic pain.^[^ ^1^, ^23^, ^24^, ^25^, ^26^ ^]^ The adverse events associated with such commercial meshes stem from the high stiffness and the collapsing of large pores (>1 mm) under tension in the dynamic environment of the pelvic floor that pique undesirable FBR and removal surgeries owing to their non‐degradability.^[^ ^13^, ^27^ ^]^ In addition, high stiffness causes stress shielding,^[^ ^13^ ^]^ resulting in a vaginal tissue‐implant mismatch, abnormal tissue homeostasis, and subsequent pore collapse, which reduces void spaces within the mesh by more than 55% in both longitudinally and transversely implanted meshes. Combined, these lead to a significant loss of mesh‐tissue interface and restricted flow of nutrients, leading to the formation of fibrotic scar tissue.^[^ ^27^, ^28^, ^29^ ^]^ Clinically, about 10% of PP‐implanted POP patients experience symptomatic mesh complications, including vaginal exposure, erosion into the urinary tract or bowel/rectum, and organ perforation due to increased inflammation, poor tissue integration, and fibrotic encapsulation.^[^ ^27^, ^30^ ^]^ Even after tertiary expert management of mesh complications, the physical and mental effects of this experience can be life‐altering and extremely disabling.^[^ ^31^ ^]^

The widespread withdrawal of PP meshes has resulted in a significant treatment gap impacting the well‐being of millions of women, leading to an urgent critical health crisis. With a view to developing alternative meshes, this study considers the vaginal tissue micro‐environment comprising subepithelial fibrous collagen and the distribution of smooth muscles within the layers called lamina propria and muscularis, respectively. These vaginal tissue elements are crucial to conferring active and dynamic support of pelvic structures, and when damaged, can lead to the development, worsening, or recurrence of POP.^[^ ^32^, ^33^, ^34^ ^]^ In addition, the mechanical strength of the transvaginal mesh is essential during reconstructive POP surgery to maintain the architectural integrity of the fabricated meshes for restoring biophysical cues to modulate the inflammatory response in the body whilst reducing the risk of anatomical recurrence. The vagina involves 11 different cell types^[^ ^35^ ^]^ within a dense connective fibrillar connective tissue^[^ ^36^ ^]^ that may alter with birth injury^[^ ^37^ ^]^ or chronic disorders such as POP.^[^ ^38^ ^]^ Recent evidence suggests POP involves cross‐communication between non‐immune cells like fibroblasts; and immune cells, macrophages,^[^ ^35^, ^39^ ^]^ within the vaginal micro‐environment. Following implantation, similar cross‐talk also orchestrates FBRs and influences the fate of vaginal grafts.^[^ ^25^, ^40^, ^41^, ^42^ ^]^ Therefore, from a clinical perspective, it is vital to design biomaterials that can mimic tissue micro‐environment, providing initial support with immunomodulatory properties that facilitate reparative processes and minimise undesirable side effects without an onerous pathway to translation.

Recently, we reported on the application of 3D printed degradable meshes in urogynaecology using computer‐aided melt electrowriting (MEW) that enables the designing of customised biomimetic constructs using a continuous polymer jet that produces microfibers with diameters between 0.25 and 60 µm.^[^ ^1^, ^43^ ^]^ While our optimised 3D printed MEW process produced mechanically robust degradable meshes,^[^ ^15^ ^]^ these meshes elicited a higher acute inflammatory FBR than desired. The FBR was characterised by predominantly CCR7^+^ M1 macrophages that required mitigation with therapeutic SUSD2^+^ mesenchymal stem cells (MSC), mainly to mediate immunomodulatory response by polarising macrophages and increasing anti‐inflammatory M2 phenotype in vivo. However, the urgent need for alternatives in the field necessitates the exploration and generation of cell‐free bioengineered vaginal implants for clinical translation. To this end, we fabricated degradable MEW vaginal implants in a multilayer fibrous architecture comprised of variable angle‐dependent fiber alignment and hierarchical porosity and studied them in a pre‐clinical rodent subcutaneous incision and ovine vaginal implant model. Optimised implant geometry is anticipated to improve its tensile properties while maintaining highly attuned biomimetic fibrous architecture with the correct fiber angle and alignment to maximise vaginal tissue integration, cell infiltration, and neo‐collagen formation producing a favourable tissue‐implant micro‐environment with limited FBR.

The objective of the study was to determine the in vivo fate and define the optimal design geometry of the degradable alternative MEW meshes. We hypothesised that the geometric design parameters such as angle and porosity significantly impact the in vivo FBR encapsulation of the mesh and influence FBR. Angulation and porosity in the layer‐by‐layer hierarchical stacking were optimised in computer‐assisted MEW mesh fabrication with reproducibility independent of therapeutic cells. Two pre‐clinical animal models, subcutaneous mice, and vaginal sheep were used to assess the mesh‐tissue attributes following POP surgery, namely degradation, tissue integration, and FBR under a clinically relevant loading condition. Vaginal surgeries are challenging in rodent models owing to the small size of the organ; therefore, we used our established vaginal sheep model of POP^[^ ^44^, ^45^, ^46^ ^]^ to validate results found in the subcutaneous mice model. Innate immune response to MEW 3D meshes, specifically macrophage polarisation such as CCR7^+^ M1 inflammatory and CD206^+^ M2 anti‐inflammatory phenotype and tissue integration, was highlighted in both animal models. The layer‐by‐layer mesh design strategies, along with the fiber deposition pattern and pore size, altered the biomechanical stiffness of the mesh‐tissue complex generating variable bioactive architecture, which was assessed in vivo to select the potential candidate for POP surgeries. This study contributes to a deeper understanding of in vivo FBR response which is pivotal to developing novel, effective therapies for women. It further highlights that simple additive‐free strategies can suppress fibrotic encapsulation and FBR in the host tissue while driving favourable immunomodulatory response and tissue integration, that can ultimately mitigate mesh adversities such as erosion and pain.

## 2 Results

### 2.1 MEW Mesh Design and Architecture

**Figure 1:**
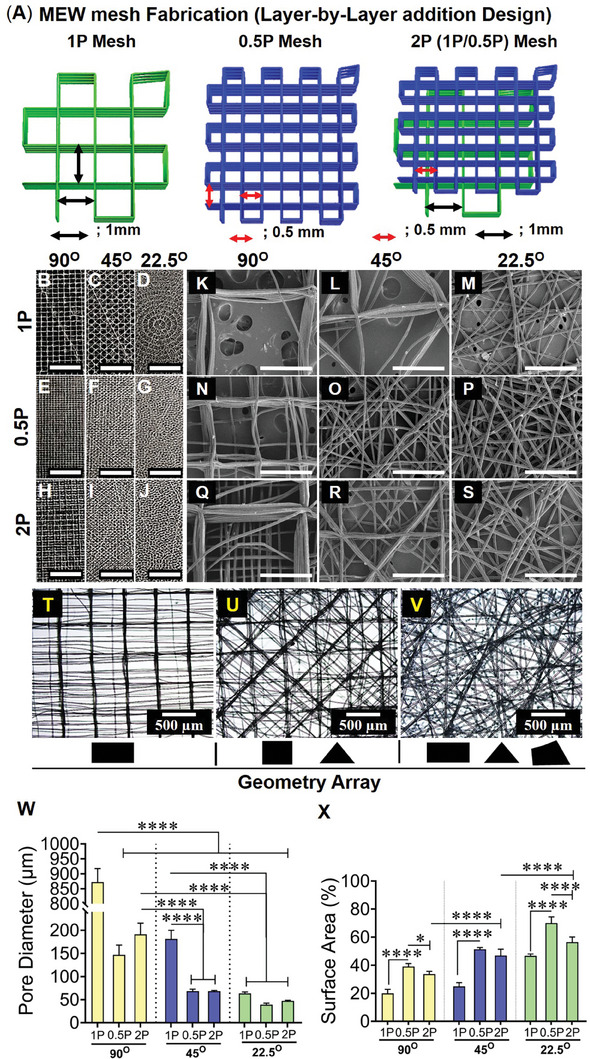
Fabrication of MEW meshes with an array of geometries and biophysical characterisation of hierarchical MEW meshes showing A) schematic of representative hierarchical mesh fabrication at 90°, B–J) mesh architecture/gross morphologies (scale bars are 5 mm) K–S) SEM images of mesh morphologies with fiber deposition, open pores, and MEW surface (scale bars are 500 µm), (T‐V) geometric arrays of the 2P structured mesh printed at 90°, 45°, and 22.5°. MEW mesh attributes showing (W) open pore diameter and (X) normalised MEW surface area. Data are mean ± SD for n = 5 meshes/group. Statistical analysis is one‐way ANOVA with Tukey's multiple comparisons test, (*p < 0.05; ****p < 0.0001).

The hierarchical mesh fabrication (i.e., 90°) combining 25 layers each of 1 and 0.5 mm inter‐fiber spacings denoted as “1P; 0.5P; 2P (1P/0.5P)” and the angle between the printed layers are shown in the representative schematic **Figures** **1**A and S1A (Supporting Information). The layer‐by‐layer addition of the overlapping of MEW fibers was greatly reduced with the construct printed at the lowest 22.5° interlayer angle (Figure S1B, Supporting Information). The gross morphology of the MEW meshes showed distinct geometric patterns (Figure 1B–J) corresponding to the interlayer angulation between 90° and 22.5°. A distinct surface topography with morphological change was observed as the angle changed from 90° to 22.5° at 1 mm inter‐fiber spacings (Figure 1B–D), which became more compacted at 0.5 mm inter‐fiber spacings (Figure 1E–G). The lowest angular constructs (Figure 1D,G,J) showed the most densely packed fibers resembling a fibrous architecture across the whole surface of the construct compared to the other MEW fabricated constructs at 90° and 45°. The layer‐by‐layer deposition of the continuous fiber jet at the set parameters is revealed by electron microscopy in Figure 1K–S. The highest overlapping of the fibers was seen in the MEW constructs printed at 90° and 45° (Figure 1K,N,Q) and (Figure 1L,O,R), respectively; in contrast, the least overlapping was observed in the 22.5° MEW printed meshes (Figure 1M,P,S). The compactness of the MEW fibers in the printed layers increased as the angle changed from 90° to 22.5°.

The higher angular hierarchical stacks revealed an array of different geometries (Figure 1T,U) generated by the combination of 1P and 0.5P meshes, wherein 90°2P and 45°2P demonstrated mostly rectangles and triangles similar to non‐hierarchical 1P and 0.5P meshes of the same angle (Figure 1B,C,E,F). In 22.5° meshes, particularly 22.5°2P meshes, the ordered rectangle or triangle geometries were replaced by the fibrous architecture including polygonal corners (Figure 1V). The lowest angular construct, 22.5°2P angular setting generated a complex array of geometries with small angles. All individual 3D printed fibers had an average diameter of 18.86 ± 2.1 µm (Figure S1C, Supporting Information) and the overall average thickness of all MEW meshes was 326 ± 59 µm (Figure S1C, Supporting Information). The weight per volume of the 50‐layer constructs was consistent among all MEW mesh groups (Figure S1D, Supporting Information). Given the greatest overlapping of the fibers was revealed in MEW meshes printed at 90° for all types and 45°1P meshes, the geometries resulted in repeated stacking in a vertical direction producing the largest open pore diameter (872 ± 45.48, 147 ± 21.05, 191 ± 76.15, and 182 ± 18.48 µm) for 90°1P, 90°0.5P, 90°2P and 45°1P, respectively, compared to other meshes (Figure 1W). The least open pore diameter (63.5 ± 2.9, 39.2 ± 3.1, and 47.3 ± 4 µm) was measured for 22.5°1P, 22.5°0.5P, and 22.5°2P meshes, respectively. Due to the least overlapping, the stacking of the fiber layers for both 45° and 22.5° printed meshes had higher surface areas than the 90° meshes for all 1P, 0.5P, and hierarchical 2P meshes (Figure 1X). In each type of printed mesh in the different groups, the reproducibility of the design patterns was extremely high as visualised by the small standard deviations in Figure 1W–X and Figure S1C,D (Supporting Information). These results show that the smallest 22.5° angular meshes can attribute optimal pore size and enhanced bioactive surface which can be beneficial for tissue regeneration.

### 2.2 Assessment of Mechanical Properties of MEW Mesh Under Tensile Loading

**Figure 2:**
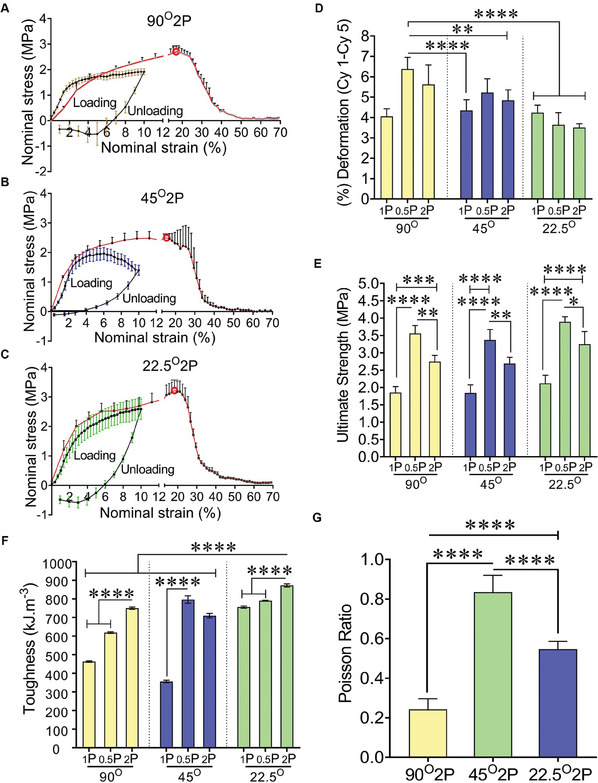
Mechanical characterisation of MEW meshes under uniaxial tensile loading at dry conditions showing stress‐strain curve (A–C) 1st cyclic loading‐unloading cycle (black solid line) and under increasing tensile loading before failure (red solid line) for 90°2P, 45°2P and 22.5°2P MEW meshes; red bordered white dots are the rupture points, D) (%) deformation after a five loading‐unloading cycle, E) ultimate strength, F) toughness of MEW meshes assessed by monotonic stress‐strain curve before failure and G) Poisson's ratio of MEW 2P meshes. Data are mean ± SD for n = 5 meshes/group. Statistical analysis is one‐way ANOVA with Tukey's multiple comparisons test, (*p < 0.05; ***p < 0.0004; ****p < 0.0001).

Preliminary mechanical properties of MEW meshes were assessed under 5 consecutive cyclic loading‐unloading and monotonic tensile loading before failure in dry conditions (**Figure** **2**A–C; Figure S2A–F, Supporting Information). During cyclic loading, 22.5°2P meshes exhibited the greatest strength (2.6 MPa) at 10% nominal strain (Figure 2C). The inelastic deformation under the cyclic tensile loading was 3.5% in 22.5°2P meshes between cycle 1 and cycle 5 (Figure 2D). In contrast, 90° and 45° meshes exhibited the least strength (1.6‐2 MPa) (Figure 2A,B; Figure S2A–F, Supporting Information) with an average inelastic deformation of 4.8% (Figure 2D). 0.5P MEW meshes had the greatest elastic modulus (19.5–35.4 MPa) and 1P MEW meshes had the least stiffness (1.2 N mm^−1^) (Table S2, Supporting Information). Hierarchical 22.5°2P meshes demonstrated moderate elastic modulus and stiffness (16 MPa, 2 N mm^−1^). All MEW meshes exhibited inelastic deformation under tensile loading at 10% nominal strain, for which the work done was calculated to assess the comparative potential of the MEW meshes (Figure S2G, Supporting Information). 22.5°0.5P and 22.5°2P meshes showed the greatest work‐done across cycle 1‐cycle 5, wherein a 57% reduction was measured compared to those of 90° and 45° meshes with 70% reduction. The inelastic deformation of the meshes resulted in the original length increase of the meshes before any failure. In effect, higher buckling loads (negative stress) were exhibited in all 0.5P MEW and greater load‐sustaining 90°2P and 22.5°2P meshes during the unloading cycles (Figure 2A–C; Figure S2A–F, Supporting Information). Under the monotonic tensile loading before failure, the corresponding maximum load before rupture (Figure S2K, Supporting Information) and the ultimate strength of the meshes (Figure 2E) were assessed at 70% nominal strain. The meshes with the least porosity (0.5P) meshes had the maximum load‐bearing capacity, with the greatest ultimate strength observed in 22.5°0.5P meshes (3.8 MPa; 19.1N) (Figure 2E; Figure S2K, Supporting Information). However, 0.5P meshes sustained the least tensile strain (avg 12.14%) compared to 1P meshes (avg 13.9%) and 2P meshes (avg 17%) (Figure S2H, Supporting Information). Among 2P hierarchical meshes, 22.5°2P meshes demonstrated the greatest maximum tensile load (18.3 N) at 18% nominal strain with an ultimate strength of 3.2 MPa. Moreover, 22.5°2P meshes also exhibited a higher toughness (Figure 2F) than 90°2P meshes (87, 75 kJ m^−3^) and 45°2P meshes (71 kJ m^−3^). The extent of lateral contraction and subsequent pore‐collapsing was assessed by Poisson's ratio (Figure 2G), showing the greatest lateral contraction of 45°2P meshes (0.8). The lateral contraction was significantly minimised in 90°2P and 22.5°2P meshes with Poisson's ratio of 0.2 and 0.6, respectively. The dry‐state mechanical characteristics of the meshes were compared with human vaginal tissues obtained from severe POP patients (Figure S2, Supporting Information). In contrast to MEW meshes, the prolapsed tissues sustained 70% elongation without rupture. At 70% nominal strain, prolapsed tissues exhibited the least tensile strength (0.56 ± 0.3 MPa) with a tensile force of 13.4 ± 7.2 N and the least stiffness of 0.18 N/mm. All MEW meshes exhibited greater tensile strength (1.8‐3.8 MPa) along with the least variability (Figure S2I, Supporting Information) compared to the prolapsed tissues.

### 2.3 Surface Topography and Cellular Interaction

**Figure 3:**
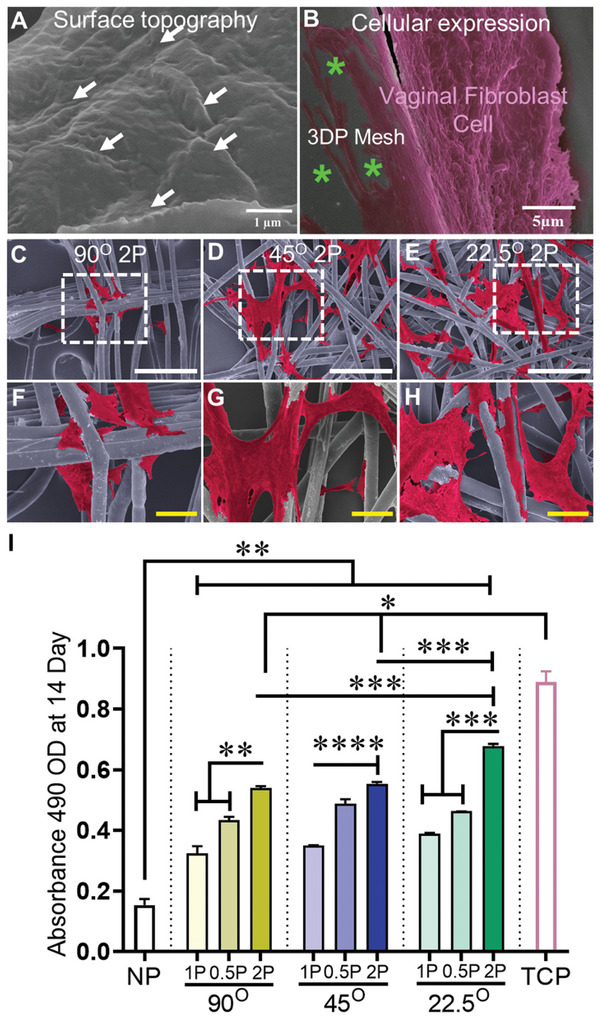
Surface topography and cellular interaction showing A) nanoscopic features (white arrows) facilitating B) the spreading of the cytoskeleton (pink color) of vaginal fibroblast on 3DP MEW mesh (green asterisks), C–H) SEM image of vaginal fibroblast's attachment (cell cytoskeleton; red color, white scale bars are 500 µm and yellow scale bars are 50 µm) and I) their proliferation at day 14 of vaginal fibroblast cells from non‐POP patients. NP represents “no pore” mesh, and TCP represents “tissue culture plate”. Data are mean ± SD. Statistical analysis is two‐way ANOVA with Tukey's multiple comparisons tests, (*p < 0.05; **p < 0.009; ***p < 0.004, ****p < 0.0001).

The biophysical cues of the printed MEW meshes were assessed for interactions with vaginal fibroblasts in vitro. MEW meshes exhibited a nanoscale surface topography (**Figure** **3**A, white arrows) interacting with VFs (Figure 3B). The angulation and porosity of MEW meshes due to the layer‐by‐layer addition of MEW process showed a distinct in vitro cellular attachment of VFs derived from non‐POP patients (age 67.6; SD 16.6 in Table S3, Supporting Information) (Figure 3C–H). The smaller angular meshes, specifically, hierarchical 45°2P and 22.5°2P meshes, had apparent visual differences in the number of VFs at day 7 and day 14, wherein the lowest angle, 22.5°2P meshes, showed the greatest number of cells (Figure S3A–C, Supporting Information; Figure 3C–H). The angulation in the mesh design resulted in significant proliferation (*p* < 0.009) of vaginal fibroblasts compared to the no‐pore meshes at days 1, 7, and 14 (Figure 3I; Figure S3D–E, Supporting Information). On days 1 and 7, the smaller angular 45°2P and 22.5°2P meshes demonstrated an upward trend in VF proliferation compared to other meshes. The proliferation of vaginal fibroblasts along with their cell‐cytoskeleton areas (Figure 3F–H) from healthy non‐POP patients was significantly increased (*p* < 0.0001) on the least angular 22.5°2P constructs at day 14 compared to meshes printed at 45° and 90° (Figure 3I). This indicates that the smallest 22.5°2P constructs with optimal angulation and porosity can attribute a significant VF attachment and proliferation in vitro, which can be beneficial for tissue regeneration in vivo.

### 2.4 Assessment of MEW Meshes’ Fate Following Implantation in Mice Model

**Figure 4:**
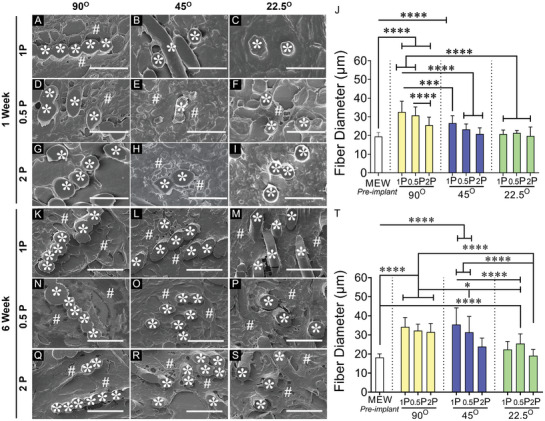
MEW mesh fate and histological assessment of the meshes after 1 and 6 week implantation in mice model showing (A–I) and (K–S) SEM images of the explanted meshes in cross‐section. MEW mesh fibers are represented by white asterisks (*) and collagen deposition is represented by white hash (#), J,T) fiber diameter in vivo after 1 and 6 week respectively. Scale bars are 50 µm. Data are mean ± SD, n = 6 meshes/group. Statistical analysis is one‐way ANOVA with Tukey's multiple comparison test, (*p < 0.05; ***p < 0.0003; ****p < 0.0001).

Given the ester group in the polymeric chain, the degradability of PCL‐based MEW meshes can be associated with fiber deformation (diameter‐increase) by water uptake/hydrolytic swelling called hydrolytic mechanism.^[^ ^47^ ^]^ The hydrolytic swelling/water uptake, thereby degradability of MEW meshes, was assessed for up to 4 weeks in vitro (Figure S5, Supporting Information) and 6 weeks in vivo (**Figure** **4**; Figure S6, Supporting Information). In vitro, the dry weight of 9 MEW meshes after submerging into PBS at 37 °C was consistent up to 3 weeks. At 4 weeks, there was a downtrend of MEW meshes (Figure S5, Supporting Information) correlating hydrolytic swelling, thereby, degradation after prolonged PBS soaking. The cross‐sectional diameter of the pre‐implant meshes revealed a similar diameter range; 16–24 µm; (mean, 19.5 ± 2.2 µm) (Figure S6A,B, Supporting Information) to original mesh diameters observed under SEM 18–20 µm; (mean, 18.8 ± 2.2 µm) (Figure S1C, Supporting Information). Ex vivo, a greater abundance of higher diameter were revealed in higher angular 90°2P and 45°2P meshes ranging from 22–32 µm compared to the smallest angular 22.5°2P meshes with a diameter range of 12.6–26 µm (Figure S6C–H, Supporting Information). The fiber diameter was significantly increased ex vivo in 90° MEW meshes compared to small angular 45°, 22.5°MEW meshes (Figure 4J,T), with swelling to ≈65% at 1‐week implantation (33 and 31 µm, respectively, for 90°1P and 90°0.5P) and was significantly reduced (*p* < 0.0001) in the 2P structured meshes with 45°, 22.5° angular deposition. After 6 weeks, the diameters were significantly increased to 27% for 45° MEW meshes and 10% for 90° MEW meshes. The hydration was significantly less pronounced (*p* < 0.0001) in the smallest 22.5° MEW meshes at 1 and 6 weeks. The least fiber swelling in all 22.5° meshes referred to the least degradation (*p* < 0.0001) compared to the higher angular 90°, 45° MEW meshes. In addition to the water uptake/swelling of MEW meshes, the hydrolytic degradation of MEW meshes was correlated with the phagocytosis revealed by the recruitment of multinucleated giant cells (MGCs), thereby, host‐tissue‐dependent foreign body reaction (FBR) following mesh harvesting in vivo. The recruitment of MGCs was qualitatively assessed (Figure S7, Supporting Information), to show the impact of mesh's geometry directing phagocytosis of MEW meshes in vivo. The infiltration of MGCs (observed by H&E stained nuclei in and around mesh fibers) (Figure S7, Supporting Information) exhibited an acute inflammation associated with collagen encapsulation. The collagen encapsulation areas around MEW fibers exhibited the greatest spreading and distribution of MGCs in the MEW meshes printed at 90° and 45° (Figure S7A,B,D,E,G,H,J,K,M,N,P,Q, Supporting Information). In contrast, with the lowest angular meshes at 22.5°, the recruitment and formation of MGCs were apparently reduced in 1 week explants and further reduced in 6 week explants (Figure S7C,F,I,L,O,R, Supporting Information). The greatest overlapping/stacking in the fiber deposition of the mesh‐fibers of 90°1P, 0.5P, and 2P meshes (Figure 4A,D,G,K,N,Q, Supporting Information) at 1 and 6 week influenced the neo‐collagen deposition around the mesh‐fibers (Figure 4; Figure S8, Supporting Information).

In 90° angular meshes, the neo‐collagen deposition seems to be deposited around the fiber‐stack (Figure 4A,D,G at 1 week and Figure 4K,N,Q at 6 week) which was more pronounced for 1P and 0.5P structures and less prominent for 2P structures. The neo‐collagen deposition was confirmed in high‐power SEM images (Figure S8, Supporting Information) at 6 week, exhibiting the deposition of collagen fibrils within the collagen bundles in and around implanted MEW meshes (Figure 4). Reducing the angular alignment of the meshes to 45° and 22.5° minimised the extent of thick collagen. Our data showed that hydrolytic swelling/water uptake may correlate with cellular interaction, which was revealed in the diameter increase of the corresponding meshes and neo‐collagen deposition (Figure 4; Figures S7 and S8, Supporting Information).

### 2.5 Fibrosis and Integration of Mesh with Host Tissue

**Figure 5:**
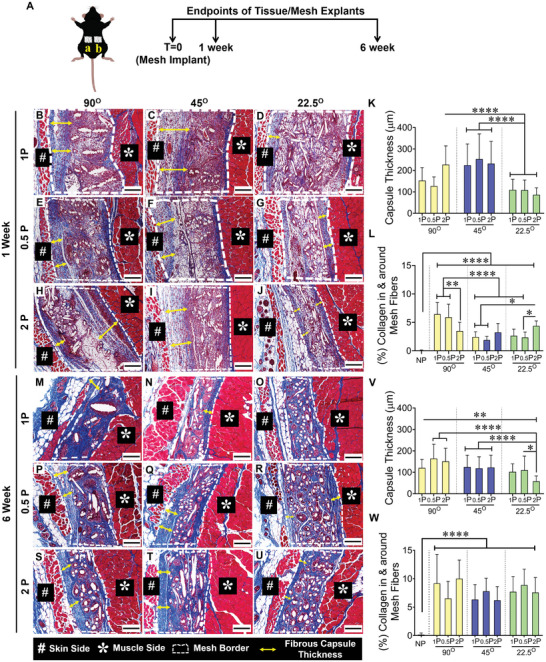
Schematic of subcutaneously implanted mesh and histomorphology assessment of fibrotic capsule thickness of MEW meshes after 1 and 6‐week implantation using Masson Trichrome staining showing mesh implantation A (a, b), the mesh tissue interface (white border), fiber alignments, and capsule formation (yellow double head arrow) after 1 week (B–J) and 6 weeks (M–U). Scale bars are 200 µm. The fibrotic capsule thickness is quantified in (K,V). The newly formed collagen area percentage inside and around the mesh is quantified in (L,W). Data are mean ± SD, n = 6 meshes/group. Statistical analysis is one‐way ANOVA with Tukey's multiple comparisons test, (*p < 0.05; **p < 0.0035; ****p < 0.0001).

MEW meshes, along with the distinct architectures based on angulation and porosity, were investigated for host tissue‐driven FBR after harvesting them from the subcutaneous pocket of C57BL6 mice (**Figure** **5**A; schematic) for 1 and 6 weeks. The MEW meshes in vivo were well tolerated and no significant changes in weight were revealed (Figure S4, Supporting Information). The foreign body response to the implanted MEW meshes was further assessed in Masson's Trichrome stained sections to precisely quantify the fibrotic response after 1 and 6 weeks (Figure 5B–W). The appearance of the fibrotic tissue in healthy skin without meshes (negative control, Figure S9A,B, Supporting Information) was used to determine capsule thickness for MEW meshes. The fibrotic response to the non‐porous solvent cast bulk scaffold sheet was used as a positive control which generated a vigorous FBR at the mesh tissue interface (Figure S9C,D, Supporting Information) leading to a substantially thicker capsule (520 µm; Figure S9D, Supporting Information). The capsule thickness for the overlapped MEW constructs at 90° and 45°, was significantly larger at both time points (Figure 5K,V, Supporting Information) compared to the least overlapping 22.5° 2P structured meshes (*p* < 0.0001).

At 1 week, when the FBR is first activated, the capsule thicknesses for all 3 mesh types (1P, 0.5P, 2P) with 90°, 45° and 22.5° patterns were 64–309, 93–450, and 39–192 µm, respectively. At 6 weeks, the FBR capsule had partially subsided with reduced thicknesses in the three groups of 53–257 µm, 49–258 µm, and 30–180 µm, respectively (Figure 5K,V). Amongst the hierarchical 2P structural groups, the 22.5°2P printed meshes generated the thinnest capsule thickness (86 µm and 58 µm at 1 and 6 weeks, respectively) compared to the other meshes. The greater percentage of collagen deposition in and around 22.5°2P meshes at 1 week (Figure 5L) combined with the thinnest capsule (Figure 5K) indicates favourable tissue integration, whereas, the greatest percentage of collagen associated with 90°1P and 90°0.5P meshes combined with a thicker capsule implies a fibrotic tissue integration. Similarly, at 6 weeks, the uptrend in collagen deposition (Figure 5W) combined with the thinnest capsule thickness (Figure 5V) for 22.5° MEW meshes indicates successful tissue integration compared to MEW meshes at 90° and 45°.

### 2.6 Macrophage Polarisation Response to MEW Meshes in Mice Model

**Figure 6:**
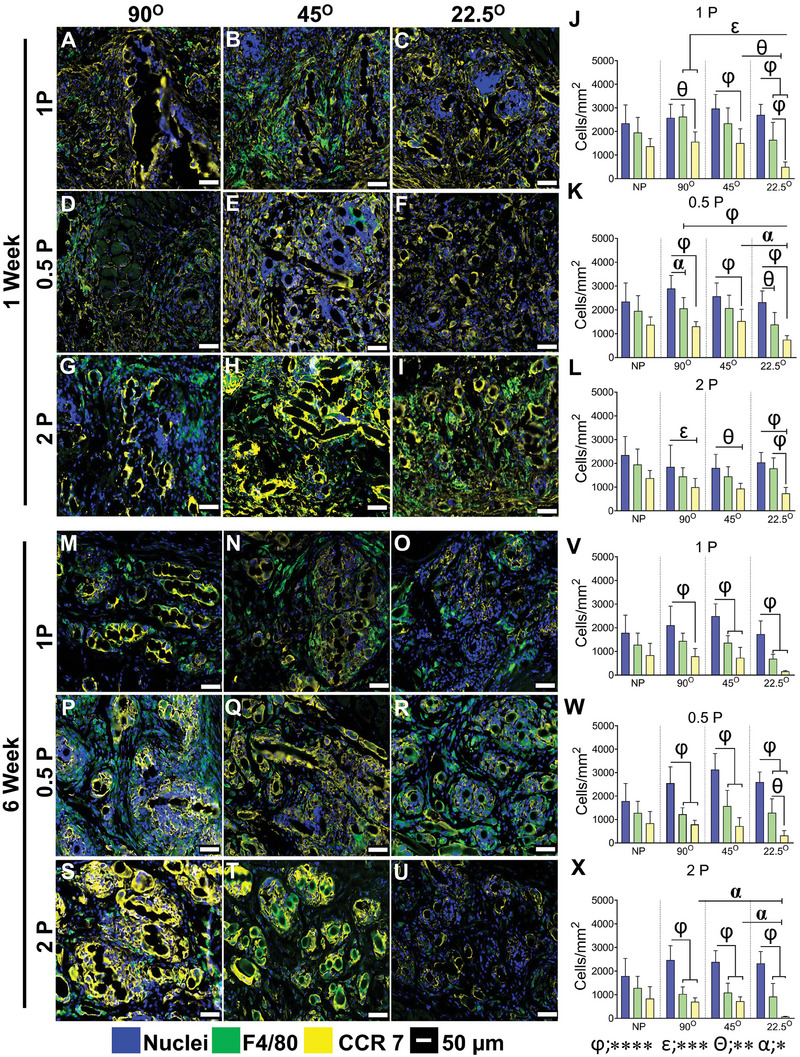
Pro‐inflammatory M1 macrophage‐associated foreign body response in mice after 1 and 6 weeks showing total macrophages immunostained with F4/80 (green, pan‐macrophage, M0); CCR7 (yellow, pro‐inflammatory M1 macrophages), nuclei (blue) after 1 week (A‐I) and 6 weeks (M–U). The number of immunostained cells/mm^2^ is shown in (J–L) at 1 and (V‐X) at 6 weeks. Data are mean ± SD, n = 6 meshes/group. Statistical analysis is two‐way ANOVA with Tukey's multiple comparisons test, (*p < 0.05 (α); **p < 0.0016 (θ); ***p < 0.0003 (ε); ****p < 0.0001 (φ)).

**Figure 7:**
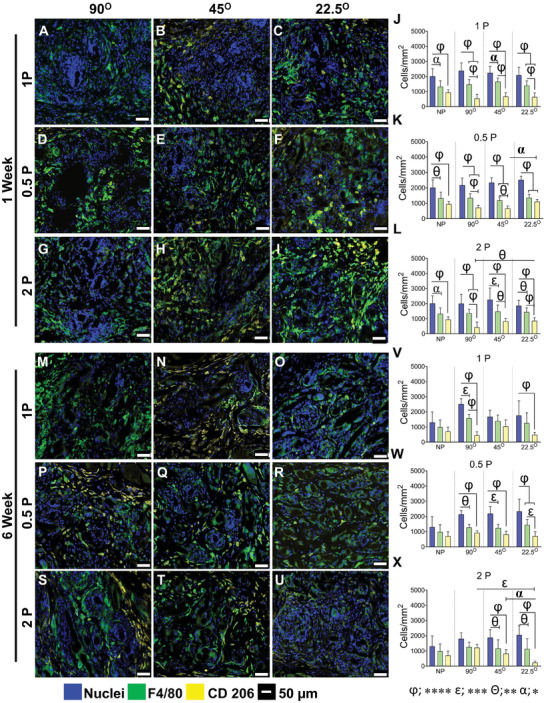
Anti‐inflammatory M2 macrophage‐associated foreign body response in mice after 1 and 6 week, showing total macrophages immunostained with F4/80 (green, pan‐macrophage, M0), CD206 (yellow, anti‐inflammatory M2 macrophages), nuclei (blue) after 1 week (A–I) and 6 week (M–U). The number of cells/mm^2^ is shown in (J–L) at 1 and (V–X) at 6 week. Data are mean ± SD, n = 6 meshes/group. Statistical analysis is two‐way ANOVA with Tukey's multiple comparisons test, (*p < 0.05 (α); **p < 0.0016 (θ); ***p < 0.0003 (ε); ****p < 0.0001 (φ)).

Pro‐inflammatory CCR7^+^ M1 macrophages and anti‐inflammatory CD206^+^ M2 macrophage phenotypes associated with the FBR to the implanted meshes at 1 and 6 week implantation are shown in **Figures** **6** and **7** and Figure S10 (Supporting Information). The total cellular infiltration, indicated by Hoechst‐stained nuclei within the mesh area was similar in all mesh types at both time points, Figure 6J–L,V–X. However, the greatest influx of macrophages, indicated by the F4/80 marker, was observed at 1 week.

Our results show that the overall number of macrophages was similar in all mesh types (Figure 6J–L). Interestingly, the number of CCR7^+^ M1 pro‐inflammatory macrophages was significantly less in most meshes printed at the low 22.5° angle compared to the higher angle meshes at 1 week, and by 6 weeks this was further reduced in the 1P and particularly the 2P low angle meshes. The percentage of CCR7^+^ M1 macrophages in the high angular meshes printed at 90° and 45° was always higher than 50%, sometimes as high as 80%, while the opposite was evident in the lowest angular printed 22.5° meshes with values of 50% and much lower. This suggests that most of the macrophages recruited to the site polarise to M1 phenotype. These macrophages remain around the capsule of the 90° and 45° meshes and around individual mesh fibers, expressing pro‐inflammatory maker CCR7, which leads to the fibrotic tissue as observed. This is visualised in the heat map (Figure S11A, Supporting Information) with dark black indicating the lowest M1 levels.

Anti‐inflammatory CD206^+^ M2 macrophages were quantified for all MEW mesh types after 1 and 6 weeks. Similar to the above, the level of cellular infiltration within the mesh area was comparable among all mesh types at both time points. After 1 week, the percentage of CD206 macrophages was 37%, 40%, and 46% of total pan‐macrophages (F4/80+ M0) for 90°1P, 45°1P and 22.5°1P meshes, respectively. This number increased to 75% after 6 weeks for 45°1P mesh and 28% and 35% for 90°1P and 22.5°1P meshes (Figure 7J,V) and for 0.5P meshes, particularly for 22.5°0.5P MEW printed meshes compared to the other meshes (≈50%) (Figure 7K). After 6 weeks, the number of CD206^+^ M2 macrophages reduced to 30% for 22.5°0.5P, whereas it increased by ≈15% for other meshes (Figure 7W), suggesting the initial high influx of CCR7^+^ M1 have likely switched to CD206^+^ M2 macrophages. The hierarchical MEW meshes printed at 45° and 22.5° exhibited the highest percentage (~62%) of CD206^+^ M2 macrophages at 1 week, which was significantly greater than 90° 2P (≈30%) (Figure 7L). After 6 weeks, there was a change in the percentage of CD206^+^ M2 macrophages, increasing to 10% and 60% for 45°2P and 90°2P meshes, respectively and interestingly, the number decreased to 40% for 22.5° 2P meshes (Figure 7X). This is visualised in the heat map (Figure S7B, Supporting Information) with dark black indicating the lowest M2 levels.

Overall, the cellular infiltration (nuclear stain) into the MEW meshes revealed no differences at both time points. The difference (≈50%) between the total nuclei counts and total F4/80 macrophages may indicate a level of infiltrating blood monocytes, tissue‐resident M0 macrophages, or early fibroblasts. The percentage distribution of both CCR7^+^ M1 and CD206^+^ M2 macrophages suggested that the lowest angular orientation along with the hierarchical design, 22.5°2P elicited a high anti‐inflammatory response at 1 week, which had subsided by 6 weeks compared with other mesh types (Figure S11C,D, Supporting Information).

**Figure 8:**
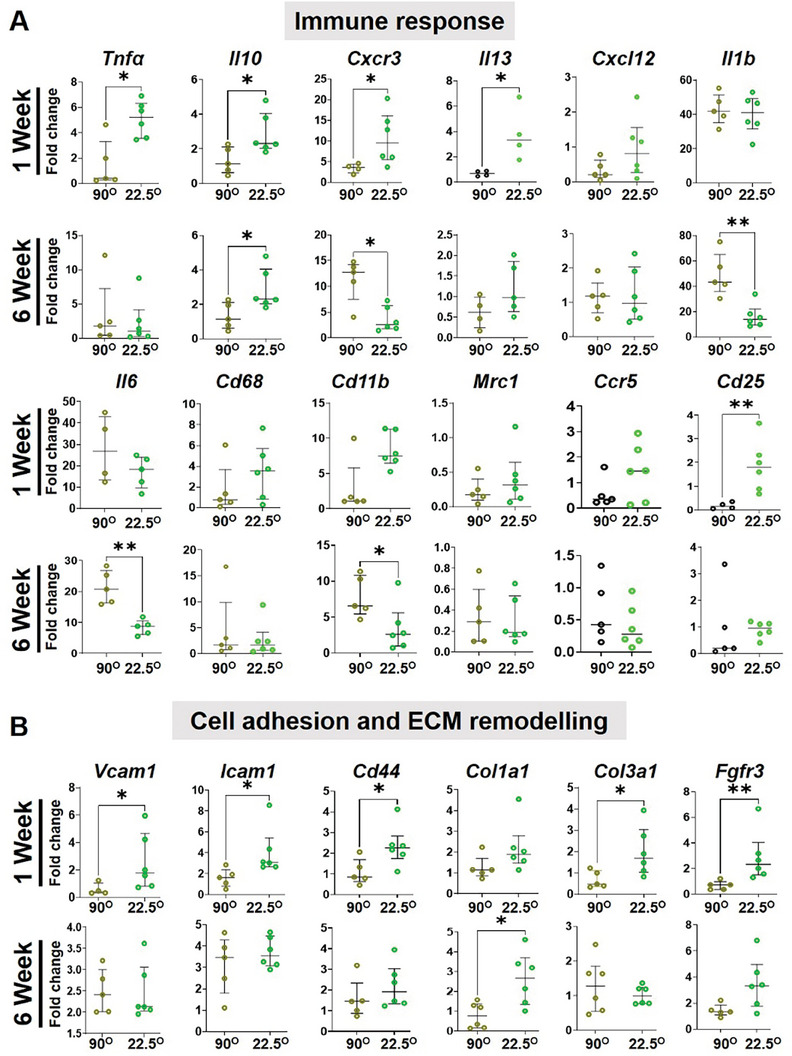
Gene expression by qPCR of tissues explanted from mice with 2P hierarchical MEW meshes after 1 and 6 weeks, showing differentially expressed genes associated with (A) immune response and (B) cell adhesion and ECM organisation indicating the impact of mesh geometry on the FBR and tissue regeneration in vivo. Data are median ± interquartile range (IQR), n = 6 meshes/group. Statistical analysis is a non‐parametric T‐test with Mann–Whitney comparison test (*p < 0.05).

Since the smallest 22.5°2P meshes modulated the immune response, enhancing tissue regeneration, they were further assessed for gene expressions compared to 90°2P meshes, (**Figure** **8**). Using the quantitative qPCR, 18 differentially expressed genes related to immune response, including cytokines and chemokines, cell adhesion, and extracellular matrix (ECM), were studied (Figure 8) to validate the recruitment of pro‐ and anti‐inflammatory immune cells along with ECM remodeling presented in Figures 5, 6, 7. At 1 week, the pro‐inflammatory cytokines including *Il1b*, *Tnfα*, *Il13*, *Il6*, and the cell adhesion markers; *Vcam1*, *Icam1*, *Cd44*, and chemokines *Cxcr3*, *Cxcl12* were mostly upregulated (*p* < 0.05) in the smallest 22.5°2P meshes which were then downregulated at the later 6 weeks time point.

In contrast, higher angular 90°2P meshes showed an upward trend at 6 weeks for cell adhesion molecules and sustained high expression for pro‐inflammatory cytokines; *Il6*, *IL1β* (*p* < 0.05), and chemokines; *Cxcr3* (*p* < 0.05), *Cxcl12*. The anti‐inflammatory molecule; *Il10* was also upregulated (*p* < 0.05) at both 1 and 6 week time points which was significantly downregulated in 90°2P meshes. The inflammation due to the foreign materials was upregulated at 1 week revealed by the uptrend of monocyte lineage gene; *CD68* and myeloid‐lineage genes; *Cd11b* in the smallest 22.5°2P which was then followed by a suppression at 6 weeks. In higher angular 90°2P meshes, *Cd11b* was significantly upregulated at 6 weeks, which correlated to the greater influx of inflammatory macrophages (Figure 6, 7). After 6 weeks, the overall pro‐ and anti‐inflammatory genes expression was suppressed in 22.5°2P meshes compared to the 90°2P meshes wherein, the pro‐inflammatory factors; *Il6*, *Il1β*, and *Cxcr3* were significantly upregulated (> fivefold) demonstrating a persistent FBR. The acute inflammation in the smallest 22.5°2P meshes at 1 week was suppressed by 6 weeks, and was associated with significant expression of tissue integration factors; *Col3a1*, *Fgfr3* (*p* < 0.05) at 1 week which then associated with *Col1a1* (*p* < 0.05) at 6 weeks. Acute inflammation due to the foreign materials was revealed by the greater expression of *Cd86* by monocytes. The smallest 22.5°2P meshes were associated with *Vcam1* expression and upregulation of inflammatory cytokines *Il1β*, *Tnfα*, and chemokine‐genes; *Cxcl12*, *Cxcr3*, *Ccr5* (Figure 8). The process also triggered simultaneous upregulation of the anti‐inflammatory marker *Il10*. This high expression correlates with the transition from M1‐proinflammatory to M2‐anti‐inflammatory macrophage presented in Figures 6 and 7. Tissue integration by M1‐M2 macrophage switching was revealed by the higher expression of *Col3a* at 1 week and *Col1a1* at 6 weeks.

### 2.7 Assessment of Vaginal Response to MEW Meshes in Sheep Model of POP

**Figure 9:**
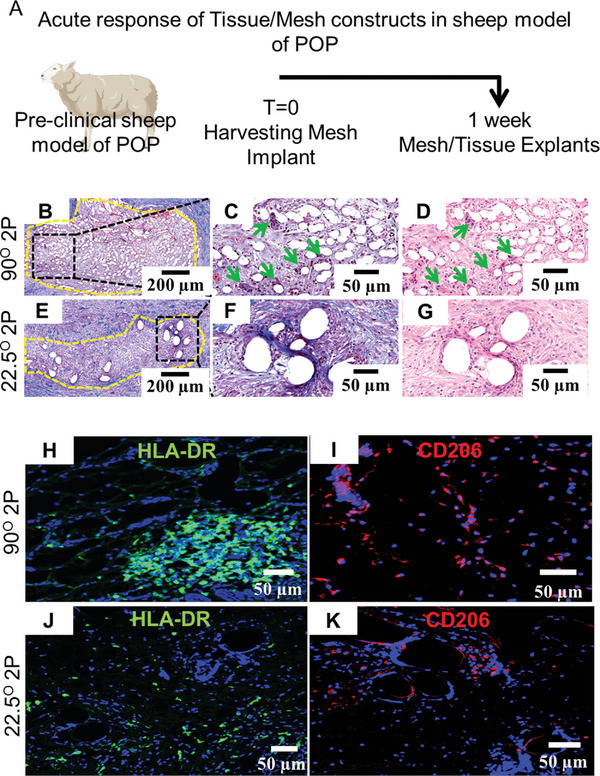
Preclinical assessment of the acute response of MEW meshes in preclinical sheep model with vaginal POP after 1 week A) Schematic of experimental timeline, B–G) acute tissue response showing the presence of multinucleated giant cells (green arrows) and neo‐collagen deposition (blue color), H,J) showing HLA‐DR expression for pro‐inflammatory M1 macrophages (in green) and I,K) showing CD206 expression of anti‐inflammatory M2 macrophages (in red) around 90° and 22.5° meshes fibers.

Considering a transvaginal application, 22.5°2P meshes were further assessed for an initial tissue integration potential compared to 90°2P meshes using our sheep model of POP.^[^ ^44^, ^48^ ^]^ After 1 week, the acute tissue response was investigated following the implantation of 22.5°2P and 90°2P meshes in the posterior transvaginal site of the sheep (**Figure** **9**). There were no signs of adversity or post‐surgical complications. Acute tissue response in vivo showed an apparently greater influx of MGCs in 90°2P mesh (Figure 9C,D) comparing 22.5°2P meshes (Figure 9F,G). Neo‐collagen deposition, thereby, tissue integration was apparently greater in 22.5°2P meshes (Figure 9E,F) comparing 90°2P mesh (Figure 9B,C). The acute tissue response associated with ex vivo 90° tissue explants obtained from sheep and mice was further compared (Figure S12, Supporting Information). Surprisingly, a similar pattern of collagen deposition was exhibited in the SEM and histologic images (Figure S12A,C,D,F, Supporting Information). Alongside, the recruitment of MGCs around the overlapped 90° meshes was similar in sheep and mice models after 1 week of implantation (Figure S12B,E, Supporting Information). Neither of the 90° and 22.5° meshes disrupted the vaginal smooth muscle with mesh fibers tightly secured by host tissue around them. Our result suggests that the collagen deposition, along with MGCs, may result in similar fibrotic capsule formation as observed in Figure 5. Due to limited antibodies available for sheep tissue analysis, antigen‐presenting marker HLA‐DR was chosen for M1 macrophage characterisation. The 90° mesh was associated with the substantial recruitment of pro‐inflammatory HLA‐DR^+^ (M1) macrophages (Figure 9H) for 90° meshes compared to 22.5° meshes (Figure 9J). Anti‐inflammatory CD206^+^ (M2) macrophages had similar expression between 90° and 22.5° meshes (Figure 9I,K). The observations in the sheep vagina reflected the findings in the mice subcutaneous tissue explants (Figures 6 and 7). Ex vivo tissue responses such as reduced pro‐inflammatory M1 macrophages and neo‐collagen deposition highlight that 22.5°2P meshes modulate the tissue response favourably.

## 3 Discussion

Chronic pelvic floor disorders such as POP have a devastating impact on the quality of life of suffering women. Reconstructive surgery is required for an estimated 20% of women,^[^ ^49^ ^]^ of which 6–36% either fail or lead to adverse events, necessitating multiple surgeries in 29% of women.^[^ ^50^, ^51^ ^]^ Lessons from the past have highlighted the importance of designing vaginal implants to mimic native tissue as closely as possible to ensure seamless integration without piquing a prolonged inflammatory FBR. Thus from a clinical perspective, this study presents an additive‐free scalable strategy to influence the resident and migrating cells in the vaginal tissue to enhance effective regeneration. Strategies utilising complex solutions such as therapeutic cells, hormones, and growth factors to achieve macrophage polarisation and subsequent extinguishing of mesh‐mediated inflammation make clinical translation onerous from a regulatory perspective. With vaginal meshes now reclassified as class III devices by regulatory bodies, an acellular tissue‐engineering strategy proposed in this study offers a promising way to augment current methods to control FBR and enhance tissue integration for faster clinical translation. Herein, we showed that FBR was influenced by immunomodulation driven by inflammatory (CCR7^+^ M1 macrophages), anti‐inflammatory (CD206^+^ M2 macrophages), and neo‐collagen deposition. Using two complementary in vivo models, the study confirmed that the polarisation of CCR7^+^ M1 inflammatory to anti‐inflammatory CD206^+^ M2 (Figures 6, 7, 8, 9) can be favourably modified by tuning of simple physical properties of the mesh that promote secretion of anti‐inflammatory factors to mitigate the formation of excessive fibrotic scar tissue.

Following the ban on non‐degradable meshes, the potential of degradable polymeric meshes is being extensively explored.^[^ ^40^ ^]^ Our study used PCL, a degradable polymer widely used in FDA‐approved products and available in medical grade for faster translation. It is widely used as a first‐choice polymer to investigate melt electrowriting parameters ensuring repeatability and scalability of the final optimal constructs.^[^ ^52^, ^53^, ^54^ ^]^ Emerging evidence suggests that degradable scaffolds also elicit inflammatory responses,^[^ ^25^, ^55^ ^]^ which can impact vaginal healing, and that strategies to mitigate them are pivotal. Recent studies have also shown the beneficial effect of therapeutic cellular tissue engineering, hormones, and extracellular vesicles^[^ ^56^ ^]^ and growth factors in combating undesirable foreign body responses.^[^ ^22^, ^37^, ^43^, ^57^, ^58^, ^59^ ^]^ This study proposed that the rational design of MEW meshes can attribute favourable FBR and tissue integration without using therapeutic cell‐based constructs. Two complementary pre‐clinical POP models were used to assess its suitability for transvaginal prolapse surgery. MEW is a rapidly expanding versatile technology that enables customisations and precise control of the design parameters.^[^ ^1^ ^]^ The prototype meshes were fabricated by MEW by varying inter‐layer angles and inter‐fiber space and characterised in the dry state to investigate the influence of mesh geometry on the in vivo FBR response and assess its suitability in pelvic reconstructive surgery. The data showed that decreasing inter‐layer angle, thereby, the inter‐fiber angle from 90° to 22.5° during fiber deposition at 1 or 0.5 mm spacing significantly alters open pore size and subsequent mesh‐surface‐area (Figure 1) beneficial for native tissue homeostasis for tissue ingrowth in vivo which is consistent with our previous findings.^[^ ^60^ ^]^ In particular, the smallest angulation, specifically 22.5°2P meshes, demonstrated bioactive surface area for greater cellular attachment, and sufficient nutrient transportation, thereby, greater tissue ingrowth within the pores. The optimal pore size (47.3 ± 4 µm) was measured for 22.5°2P meshes which was found to be optimal for tissue regenerative immune response in vivo. Unlike higher angular, 90°, and 45° meshes, the smallest 22.5° MEW meshes were associated with the least overlapping of MEW fibers that potentially suppressed FBR by forming the least foreign body giant cells with the least nuclei‐fusion. We have previously shown that the surface area of degradable meshes plays a role in influencing in vivo host cell infiltration and ultimately directing macrophage polarisation, demonstrating an additive‐free approach.^[^ ^22^ ^]^ With a simple additive‐free modification, we could critically restrict the infiltration of CCR7^+^ M1 inflammatory macrophages at the acute stage (Figure 6) yet achieve seamless tissue integration in both pre‐clinical models.

As such pelvic reconstruction is a complex surgery with several tissues involved. The forces acting on transvaginal meshes in POP repair are poorly defined and the ideal biomechanical strength requirements of degradable implants remain elusive.^[^ ^61^ ^]^ However, it is now clearly understood that a drastic mismatch between the implant properties and the vaginal tissues largely contributes to adversities leading to painful post‐operative complications and reoperations.^[^ ^62^ ^]^ The 22.5°2P meshes were lesser in stiffness compared to non‐degradable PP meshes but slightly more compared to prolapsed vaginal tissue from patients which was highly variable.^[^ ^63^, ^64^, ^65^ ^]^ As most pelvic floor implant mechanical characterisations have been done using the uniaxial method given the limitations in biopsy sizes,^[^ ^70^ ^]^ we opted for a similar method for pre‐implant tensiometry in dry conditions to characterise pre‐surgical properties. The shorter linear working range (2–4% nominal strain) of MEW meshes can be due to the shorter chain PCL (Mn 45 kDa), which could possibly be enhanced by longer chain medical‐grade PCL (Mn 120 kDa).^[^ ^66^ ^]^ The 22.5° geometry significantly increased the bioactive surface area, which is a highly favourable feature. Following surgery, physiological conditions like hydration in vivo and suturing supports, along with the neo‐collagen deposition due to the tissue‐compatible architecture such as that of 22.5°2P meshes, are likely to improve load sustenance and stiffness.^[^ ^61^ ^]^ Previously used commercial PP meshes were too strong and caused unacceptable stress shielding, leading to tissue deterioration.^[^ ^62^ ^]^ As such, the ideal biomechanical properties for transvaginal meshes remain unknown.^[^ ^67^ ^]^ However, it is well established that for successful outcomes they must integrate with vaginal tissue rather than disrupt homeostasis and avoid any FBR that could lead to complications.^[^ ^62^, ^67^ ^]^ Further, PP meshes with larger pores (>1 mm) had pore‐collapse that risks tissue disruption in the vagina.^[^ ^62^ ^]^ Herein, our 90°2P meshes demonstrated the least lateral contraction with the least working range, whereas 22.5°2P meshes revealed an improvement in the working range associated with the greatest nominal strain while minimising the lateral contraction compared to 45°2P meshes. This signifies the ability of 22.5°2P meshes to preserve structural integrity post‐surgically. Although our results suggest the scope of improvement in tensile in 22.5°, our cellular and molecular FBR response suggests a promising advancement, never reported before. The lost tissue strength in prolapsed vaginal tissue is likely to benefit from the strength of 22.5°2P meshes at the initial stage following mesh implantation to alleviate the symptoms until the regeneration process is complete given that mesh‐tissue complex strength is higher post‐implantation.^[^ ^64^, ^65^, ^66^, ^67^, ^68^, ^69^ ^]^ It is critical that no adversity or complications arise during this phase. We show that 22.5° meshes can effectively modulate FBR. The complex biomechanics of the human vagina and implants will highly benefit from design modifications to improve the mesh tensile properties, sophisticated biaxial testing that mimics more physiological conditions such as wet testing as well as bi‐pedal loading conditions for accurate validation of transvaginal surgical meshes. It is also essential to develop biological and computational models that can evaluate such biomechanical outcomes of transvaginal surgery in a time and cost‐effective way.

Vaginal tissue comprises 11 different cell types, of which more than half are vaginal fibroblasts (VFs)^[^ ^41^ ^]^ that play a pivotal role in maintaining native tissue homeostasis followed by mesh integration in vivo. Upon implantation, the mesh will likely come in contact with VFs in the vagina. In our in vitro biocompatibility studies, the most important factor was the cell proliferation capacity of the host VFs given the optimal angulation and porosity determining the outcome of mesh integration in vivo. To this end, our results showed that VFs were biocompatible with all MEW‐generated meshes associated with variable proliferation of VFs, with the low angle 22.5°2P meshes promoting significantly more cell proliferation (Figure 3; Figure S3, Supporting Information). Such bioactive attributes of 22.5°2P meshes in vitro correlated with tissue integration and neo‐collagen deposition in vivo.

The mesh‐related adverse events of the withdrawn non‐degradable polypropylene (PP) meshes are associated with pain and mesh erosion/exposure after implantation, which are likely related to chronic FBR. To overcome such adverse events, the degradable PCL has the potential to degrade in 2–4 years at no toxicity.^[^ ^70^, ^71^ ^]^ Hydrolytic degradation is one of the mechanisms of PCL‐degradation which can be boasted by cellular phagocytosis.^[^ ^72^ ^]^ In the study, we assessed hydrolytic degradation to investigate architectural stability and the inflammatory cellular response in vivo, resulting in the infiltration of MGCs and, therefore, fibrotic encapsulations around the implanted mesh. Fibrotic encapsulation has also been directly associated with pain after mesh placement^[^ ^73^ ^]^ and marks the remodeling phase of FBR, whereby fibroblasts produce fibrous collagen around the biomaterial.^[^ ^74^, ^75^ ^]^ The capsule formation around implanted biomaterials results from a cascade of responses at the implant‐host interface involving chemokine‐and‐cytokine‐mediated migration of immune cells, such as macrophages, that attempt to phagocytose the mesh.^[^ ^25^, ^57^ ^]^ However, when the mesh is large and beyond the capacity of a single macrophage to phagocytose it, several fuse to form multinucleated giant cells in an attempt to encapsulate it fully. Our results prove that meshes printed at a 90° angle and their subsequent vertical stacking were perceived as large foreign objects by the innate immune system. This leads to significantly larger fibrotic capsules populated with pro‐inflammatory macrophages for an extended period (Figure 6). Such cellular interaction also exhibited hydrolytic swelling of the stacked fibers resulting in increasing fiber diameter (Figure 3). On the other hand, reducing the printing angle to 22.5° markedly reduced the capsule thickness (Figure 5) around the mesh implant with fewer inflammatory M1 macrophages. It appears that the variable hydrolysis of PCL related to the distinct angulation pattern was related to the variable extent of multinucleated giant cell generation. Thus, 22.5°MEW meshes with fibrous architecture, low stiffness, and larger surface area exhibit a stable mesh architecture despite hydrolytic degradation and unique immunomodulatory response that significantly alters in vivo FBR and maladaptive fibrotic capsule formation while enhancing integration. In contrast, the highly overlapped 3D MEW meshes printed at (90° and 45°) exhibited similar behaviour to a non‐porous implant, piquing inflammation and thicker fibrotic capsule formation around the MEW fibers. Mirroring our mice subcutaneous results, both 90° and 22.5° mesh integrated well in the sheep vaginas exhibiting similar early encapsulation patterns.

Macrophages are the 3rd most abundant cell type in the vagina and key players in driving biomaterial‐mediated innate immune response and fibrosis.^[^ ^76^ ^]^ In our animal models, we confirmed the crucial and dynamic role of M1/M2 macrophage polarisation in the outcome of nine different MEW meshes. While pro‐inflammatory M1 macrophages were necessary during the acute stage for controlling fibrosis and enhancing tissue regeneration, the study showed that their transition to M2 phenotype is equally essential for tissue healing and mesh integration. The influx of M2 macrophages in 22.5° MEW meshes was significantly greater at 1 week (Figure 7), which was associated with a rapid healing process. To this end, the 22.5°2P meshes exhibited a significant and favourable reduction of CD206+ M2 macrophages by 6 weeks compared to 90°. The prolonged presence of M2 macrophages is problematic as it has been associated with transvaginal mesh complications in women, particularly pain and fibrosis that require excision surgery.^[^ ^14^ ^]^ In women, a pro‐inflammatory response that persists years after implantation has been associated with transvaginal mesh complications. On the other hand, although a high M2/M1 ratio may be beneficial, the proper timing of M1 to M2 polarisation is pivotal to the fate of implanted meshes in the vagina. This study is the first to report that mesh geometry can achieve balanced recruitment of both pro‐inflammatory M1 and anti‐inflammatory M2 macrophages in vivo. Several chronic diseases,^[^ ^77^, ^78^, ^79^ ^]^ including POP^[^ ^35^ ^]^ and POP treatment failure, have been associated with such extensive M2 presence. However, we mainly observed a heterogeneous ectopic cell population comprised of macrophages and fibroblasts around the MEW meshes, particularly at 6‐week time points, suggesting a critical interplay between these cells in driving fibrotic encapsulation. The higher angle mesh geometry strongly exhibited a co‐localisation of F4/80 and CCR7 markers in the fibrotic capsules, which were significantly thicker with variation in the neo‐collagen profile. Emerging evidence highlighted that biomaterial‐mediated fibrosis, especially after the acute inflammatory phase, exhibited a mixed profile of macrophages rather than discretely M1 or M2 phenotypes,^[^ ^15^, ^57^, ^58^, ^59^, ^60^, ^61^, ^62^, ^63^, ^64^, ^65^, ^66^, ^67^, ^68^, ^69^, ^70^, ^71^, ^72^, ^73^, ^74^, ^75^, ^76^, ^77^, ^78^, ^79^, ^80^, ^81^ ^]^ suggesting the involvement of complex molecular processes. Considering this, the differential gene expressions revealed the upregulation of pro‐inflammatory factors; *Il1b, Tnfa, Il13, Il6, Cxcr3, Cxcl12* and cell adhesion molecules; *Vcam1, Icam1, Cd44* at 1 week (Figure 8). Therefore, the acute inflammation due to the foreign materials triggered the influx of M1 macrophages revealed by the upregulation of *Cd68* and *Cd11β* genes. The greater influx of M1 macrophages, thereby switching into M2 type, was expressed in the smallest 22.5°2P meshes by the upregulation of the anti‐inflammatory gene, *Il10*, which was suppressed at a later 6‐week time point. Tissue integration by anti‐inflammatory macrophage switching was revealed in higher expression of *Col3a1* at 1 week and *Col1a1* at 6 weeks. Higher angulation and pore size, thereby, inter‐layer overlapping of 90° mesh fabrication is likely to prolong the acute response demonstrated in the persistent upregulation of pro‐inflammatory molecules; *Il6, Il1β*, and *Cxcr3*. The wide range of pore size up to 47 µm diameter in smaller 22.5° angular meshes (Figure 1) promoted in vivo cellular infiltration that resulted in a balanced immune response leading to greater neo‐collagen deposition while minimising the fibrotic capsule deposition. Given the similar effects in the mouse subcutaneous model as well as sheep vaginal tissue (Figure 9), it is likely that the molecular mechanisms involved in both tissue types may be alike. While further studies are pivotal to establishing their exact role and mechanisms of cell‐cell communication, our study suggests that they play a crucial role in homeostasis after implantation by aiding repair processes.

Post‐surgical complications arising from non‐degradable mesh require multiple corrective surgical interventions within a few years.^[^ ^82^ ^]^ Therefore, resorbable implants with higher in vivo longevity and tissue integration are highly attractive. In this study, the smallest 22.5° angular meshes sustained the controlled swelling of MEW fibers after 6 weeks in vivo in mice model while maintaining the highest surface area and the greatest cellular infiltration leading to neo‐tissue formation. Similar results were observed in the ovine vaginal site at 1 week indicating these meshes strongly integrate with the tissue and are unlikely to cause mesh erosion requiring subsequent mesh removal surgery. Degradable meshes likely circumvent long‐term adverse effects with degradation kinetics of PCL of around two years or less.^[^ ^52^ ^]^ Thus it is pivotal that the new generation of pelvic floor biomaterials actively interacts with tissue to promote healing and integration prior to complete degradation rather than merely having an inert presence.^[^ ^25^ ^]^ 22.5°2P meshes with greater surface area and optimal pore sizes, demonstrated a tuned degradation associated with an endogenous cellular response resulting in tissue regeneration.

### 3.1 Study Limitations

This proof‐of‐concept study is not without limitations. One limitation is using a subcutaneous mice model instead of a vaginal rodent model. However, successful mesh integration in the large subcutaneous pocket indicates likely effectiveness in the vaginal environment, which has more surrounding cells. Mouse subcutaneous implantation allows detailed analysis of cell migration using highly specific antibodies cost‐effectively. To address differences in the vaginal environment, we also confirmed our findings in a sheep model of POP, which closely resembles the human vaginal environment. Another limitation is the degradation kinetics of PCL, which requires longer evaluation periods in both models. We used uniaxial tensile testing which, while not ideal, allows comparison with other studies. A bi‐axial tester would mimic vaginal forces more appropriately. Additionally, the research‐grade PCL used here had a shorter chain length, affecting mesh yield strain; future studies with medical‐grade PCL would be necessary. There is a limited range of specific antibodies and reagents for immune response evaluations in sheep tissues. Despite these limitations, combining the two models provides valuable insights. The biomechanical stability post surgically will benefit from further evaluations in bi‐pedal models such as non‐human primates. Lastly, the pandemic lockdowns in Melbourne affected the number of animals and time points. Future studies evaluating longer time points would be critical for clinical translation.

## 4 Conclusion

With the rise in the elderly population, POP represents a significant healthcare burden without reliable treatment options. Based on the lessons learned from previously used pelvic meshes, we fabricated degradable 3D‐printed meshes using melt electrowriting to boost endogenous tissue repair following the POP progression. The mesh fabrication was investigated for optimal porosity and angulation‐dependent biophysical surface architecture. Using mice and sheep pre‐clinical animal models, we showed that a reduction in angular geometry can significantly alter FBR in host tissues by minimising fibrotic capsule formation, mitigating inflammation, and improving tissue integration. Given that FBR is a major obstacle in the long‐term success of pelvic floor reconstructive surgical biomaterials, the current MEW meshes appear as additive‐free alternatives with computer‐embedded scalable fabrication for treating POP. The data validation using two pre‐clinical models suggests the role of mesh geometry in modulating implant/tissue reaction is a pivotal step for next‐generation pelvic floor implants.

## 5 Experimental Section

### Design and Fabrication of MEW Meshes

MEW printed meshes were fabricated using a Bioscaffolder 3.1 (3D Printer) with a melt electrowriting tool head (GeSIM, Germany) installed inside a Biological Safety Cabinet. A CAD model was generated in Microsoft 3D builder and imported into Bioscaffolder software (GeSIM) for slicing and g‐code creation. For printing, Poly (*ε*‐caprolactone) (PCL) beads, average M_w_ 45 KDa (Sigma–Aldrich, Australia) were loaded into the MEW stainless steel cartridge (10 mL) with melting temperature set at 100 °C, as optimised previously.^[^ ^15^, ^43^ ^]^ Modifications to the printer included the substitution of the ‘manufacturer's non‐conductive Teflon substrate with conductive stainless steel (SS) substrate. Printing parameters; voltage (V), 4.8 KV; tip to collector distance (TIC), 10 mm; extrusion pressure (P), 5–8 KPa and printing speed, 10 mm sec^−1^ were optimised. MEW meshes were printed with 50 layers at three different interlayer fiber angles; 90°, 45°, and 22.5° with variable inter‐fiber spacings; 1 and 0.5 mm which were defined as 1 and 0.5 P respectively. In MEW mesh terminology, P stands for porosity with the prefix 1 or 0.5 informing the inter‐fiber space. The hierarchical pore gradient constructs were obtained by printing the 1st 25 layers at 1 mm spacing (1 P), followed by the 2nd 25 layers at 0.5 mm spacing (0.5 P). The hierarchical mesh was defined as 2 P indicating two interfiber spaces; 1 and 0.5 mm. In total, nine different MEW 50 layered constructs with differing angles and inter‐fiber spaces were printed and the final definition and the mesh terminologies are outlined in Table S1 (Supporting Information). A non‐porous sheet construct was fabricated using solvent casting as a negative control.

### Mechanical Characterisation and Degradation Assessment

MEW mesh morphology was observed (n = 5/group) under scanning electron microscopy (SEM) (Nova NanoSEM, FEI, USA) using an accelerating voltage of 10 KV and a working distance of 3.5 mm. Samples were prepared following the previous publication.^[^ ^23^ ^]^ SEM images were analysed to measure the diameter of the printed fibers and the thickness of the MEW meshes. Ten individual fibers per sample were measured. Open pore size and mesh surface area were quantified on three images/sample (n = 5 samples) by an Olympus BX 41 Phase Contrast Microscope and image J using the macros developed. For open pore size calculation, the “Invert” function followed the threshold setting (Figure S1E, Supporting Information). Afterward, a series of binary operations; “Despeckle”, “Remove outliers”, “Erode”, “Close” and “Analyse particles” excluding 10 µm size particles were performed. The “Results” window was saved for pore size assessment. The calculation of (%) surface area followed the operations except performing “Invert” function (Figure S1E, Supporting Information) as mentioned above and finally, was normalised to the total mesh area.

For mechanical assessments of the MEW meshes, the Univert biomaterials testing platform (CellScale, Canada) was used with 0.1 N preloading setup prior to uniaxial tensile loading in dry conditions. The platform was prepared under displacement control mode, defining the extent of the elongation limit in comparison to the initial gauge length. Therefore, resistive force per unit displacement was measured by the load cells. MEW meshes (4.5 cm × 1.5 cm) were fabricated (n = 10 meshes/group) to assess mechanical attributes under both monotonic loading (to failure) and cyclic loading regimes. Cyclic loading was conducted at 10% displacement of the gauge length for five consecutive loading‐unloading cycles, and monotonic loading was conducted at 70% displacement of the gauge length to reach the rupture point for all samples. Stress‐strain curves were derived from force‐displacement data generated by the testing platform. The cross‐sectional area for the stress calculation was obtained from the initial dimensions (thickness × width) of the printed scaffolds, therefore, the porosity of the meshes was not considered. The study reported nominal strain calculated based on the gauge length. Under cyclic loading, (%) deformation was measured by the ratio of the length increase after five consecutive cycles, and the original length before the cyclic assessment and the functionality/work done was measured by the area under the cyclic stress–strain curve. Under monotonic loading to failure, six distinct mechanical attributes including ultimate strength, toughness (measured by area under the stress‐strain curve before failure), maximum strain and corresponding maximum force before failure, and stiffness (slope derived from the force vs displacement curve in the elastic region during the loading cycle) and Poisson's ratio (negative of the transverse strain to the axial strain at the maximum tensile load) for the lateral contraction indicative of pore‐collapse. MEW meshes (1.5 cm × 1.5 cm) were further soaked in PBS (n = 5/group) at 37 °C for 7, 14, 21, and 28 days. After soaking, the meshes were dried and weighed to assess their degradation, measured by corresponding weight changes in vitro.

### Isolation and Culture of Primary Vaginal Fibroblasts (VF)

Human vaginal tissues were collected from post‐menopausal non‐POP patients (n = 5). The patient's age and the stage of POP are presented in Table S3 (Supporting Information). All human tissues were approved for use by the Monash Health Human Research Ethics Committee (09270B) and all patients gave written informed consent. Vaginal fibroblasts (VF) from non‐POP patients were isolated from biopsies. Vaginal tissues, with epithelium removed, were minced and dissociated by 5 mg mL^−1^ collagenase type I, 4 mg mL^−1^ deoxyribonuclease type I (DNAse I) (both Worthington‐Biochemical Corporation) and 5 mm Glucose at 37 °C in Dulbecco's modified Eagle's Medium/F12 medium (DMEM/F12) supplemented with 15 µm Hepes (Invitrogen) using rotation on MACS Mix (Miltenyi Biotech) for 60–90 min until single cells were released. The single cell suspension was passed through a 40 µm sieve (BD Bioscience) and red blood cells were removed by density gradient centrifugation using Ficoll‐Paque (GE Healthcare Bioscience‐BioSciences AB) and then cultured in DMEM/F12 medium containing 10% FBS (Invitrogen), 1% antibiotic‐antimycotic and 1% glutamine (both Life Technologies) to full confluency in Corning cell culture flasks. Non‐POP VFs were then seeded on the mesh (10^4^ cells/mesh type, n = 5). The cytocompatibility was assessed by MTS assay after 1, 7, and 14 days incubation using 2% MTS reagent (Promega, USA) in serum‐free DMEM medium (1 mL) for 2 h at 37 °C in 5% CO_2_. After 24 days, the cellular attachment on the distinct angulation and porosity meshes was observed under SEM with the same settings mentioned in Section 5.2. Sample preparation for SEM imaging started with 4% PFA fixation for 10 min at room temperature followed by dehydration in ethanol series (50%, 75%, 95%, and 100% for 5 min). After complete dehydration, a critical point drying agent; hexamethyldisilazane, HMDS (reagent grade ≥99% Aldrich) was applied on the mesh surface inside a biohazard suction hood overnight. After drying, samples were mounted over carbon tape and sputter‐coated with platinum particles for SEM imaging. In parallel, 4% PFA fixed samples were permeabilised with 0.1% Triton X‐100 and blocked for non‐specific staining using protein block (Dako) for 1 h at room temperature. After blocking, samples were immunostained with TRITC‐conjugated phalloidin (milipore) at 1:500 for 3 h followed by 3 washes with PBS and finally nuclei staining at 1:1500 for 3 min with same washing. Samples were imaged using a FluoView FV 1200 confocal microscope (Olympus). To observe the cell cytoskeleton with MEW mesh‐morphology, VFs seeded samples on day 14 were processed for SEM imaging. Briefly, after 4% PFA fixing of the cell‐seeded MEW meshes, samples were dehydrated in ethanol series (50%, 75%, 95%, and 100% for 5 min) that followed a critical point drying of the meshes using hexamethyldisilazane: HMDS (reagent grade ≥99% Aldrich) inside a biohazard suction hood for overnight. After drying, samples were mounted over carbon tape and sputter‐coated with platinum particles for SEM imaging.

### Surgical Implantation of MEW Meshes in C57BL/6 Mice

All experimental mouse procedures were approved by the Monash Medical Centre Animal Ethics Committee A (MMCA/2017/05). Both the surgical procedure and post‐implantation care of the C57BL/6 mice and sheep followed the National Health and Medical Research Council of Australia guidelines for the care and use of experimental animals. A total of 46 mice were randomly divided into four mesh groups including 90°, 45°, and 22.5° (total 9 meshes/group). In addition, non‐porous (NP) meshes were used as negative controls to determine the impact of angle and porosity on various tissue responses investigated in the study. The healthy controls (only skin without meshes) were used for normalising the data. For all angle groups; 90°, 45°, and 22.5°, each mouse received two identical implants on the left and right side, symmetric to the sagittal plane which accounted for n = 12 meshes for each of the three hierarchical 2P mesh groups and n = 8 meshes for each of the three 1P, three 0.5P and one NP sheet, respectively. Table S4 (Supporting Information) presents the distribution of 46 mice with the respective meshes. Meshes were weighed and sterilised under UV light (120 Watts of commercial grade 254 nm wavelength germicidal UV light, Laftech.com.au) for 30 min on each side. The sterilised meshes were pre‐soaked in stromal medium containing 1% Antibiotic–Antimycotic (Thermofisher) overnight, then subcutaneously implanted for 1 and 6 weeks. Mice were weighted and anesthetised with 3% w/v Isoflurane and Carprofen (5 mg kg^−1^ body weight) was used as an analgesic. The mice's abdomen was shaved and sterilised with 70% ethanol. An incision (1 cm) was created using sharp, sterile scissors and blunt dissection to create a subcutaneous pocket, and two single 1 × 1 cm meshes were implanted/mouse with larger pores of the hierarchical mesh facing the abdominal wall. Surgical sites were closed with a single intracutaneous Ethicon 6‐0 monofilament suture. After surgery, mice were individually housed and provided with Carprofen (0.3–0.5 mg/100 gm body weight) supplemented water. After 1 and 6 weeks, mice were weighed and euthanised by CO_2_ inhalation. The mice tissue explants were then processed for both histologic and immunohistologic assessments described in Sections 5.6 and 5.7.

### Surgical Implantation of MEW Meshes in Sheep Model of POP

All experimental sheep procedures were approved by the Monash Medical Centre Animal Ethics Committee A (MMCA/2018/30). The procedure involved pre‐screening of Monash flock of non‐pregnant sheep (parity ≥3) by vaginal POP‐Q (clinical pelvic organ prolapse‐quantification) measurement established by the group. This pre‐screening selected prolapsed sheep (n = 4 sheep in total) with POP‐Q's ≥ −1 cm at Ap (posterior) position,^[^ ^44^, ^83^, ^84^ ^]^ which implicated a posterior vaginal tissue laxity. Sheep were hormonally synchronised using intravaginal progesterone (0.3 g) releasing device for 2 weeks and removed 48 h prior to vaginal surgery as per routine pre‐operative management. On the day of surgery, mesh implants, 90°2P (n = 1 mesh) and 22.5°2P (n = 3 meshes) of 3 × 2 cm size meshes, were inserted in transvaginal sites of four sheep following the published method.^[^ ^48^ ^]^ Briefly, sheep were fasted overnight and anesthetised intravenously with Medetomidine (0.01–0.02 mg kg^−1^) and Thiopentone (10 mg kg^−1^) and maintained under Isoflurane (0–5% in 100% O_2_). Antibiotic Cefazolin (7.5 mg kg^−1^) was provided. Sheep were placed in a lithotomy position followed by hydro‐dissection of the vaginal tissue using 5 mL of Bupivacaine (5 mg mL^−1^) with 1 mL adrenaline diluted to 20 mL with saline to separate the vaginal layers. Following a vaginal incision and retraction of the tissue with a Lone Star device, 90°2P (n = 1 mesh; 1 sheep) and 22.5°2P meshes (n = 3 meshes; 3 sheep) were placed under epithelium into the vaginal wall and secured using 4‐0 Polydioxanone sutures. The vaginal epithelium was closed using absorbable 3‐0 Vicryl suture and sheep were recovered using Atipamezole (0.05 mg kg^−1^). Carprofen (2 mg kg^−1^) was administered intravenously for pain relief and sheep were monitored for 3 days post‐surgically. After 1 week, sheep were humanely euthanised with intravenous pentobarbitone (150 mg kg^−1^), and the whole vagina was explanted during post‐mortem. The regions of the mesh‐tissue complex were explanted and processed for histologic and immunohistologic assessments described in Sections 5.6 and 5.7.

### Histologic Assessment of Explanted MEW Meshes

Mesh‐tissue explants obtained from mice and sheep were fixed in 10% formalin, then processed into paraffin blocks and sectioned at 5 µm onto poly‐l‐Lysine slides. Unless otherwise stated, all steps were performed at room temperature (RT). Sections were stained with H&E and Masson's trichrome to observe histomorphology and assess tissue integration, fibrotic capsule formation, and collagen deposition. The entire cross‐section of the stained tissue was scanned using the Aperio Leica biosystem, Monash Histology Platform at 40x magnification and analysed using ImageScope [V12.3.3.5048] software. The quantification of fibrotic capsule thickness was performed in ImageJ using the scale bars obtained in ImageScope software. The macros were developed in Image J software to quantify the collagen content in and around the mesh‐tissue border. Briefly, for collagen quantification, a color deconvolution at vector “Masson Trichrome (MT)” was performed to separate the collagen deposition in and around MEW mesh explant from surrounding collagen using the “Otsu” imaging optimal thresholding algorithm to convert grey images to a binary image to separate foreground pixels (Collagen) from other tissue background elements to obtain a total collagen area as a percentage of the total area examined. For total area, the macro was programmed to allow a manual interface for drawing the region of interest (ROI) around the mesh/tissue border clearly demonstrated in MT images.

### Immunological and Gene Expression Profiling of Explanted Tissues

Pre‐implant meshes and mesh‐tissue complexes were fixed in 4% PFA and processed into OCT frozen blocks which were cryosectioned (5 µm) on both bare poly‐l‐Lysine slides and on aluminum foil‐wrapped slides. The diameter of the mesh fibers was measured from cross‐sectional mesh/tissue slices obtained from OCT‐embedded cryo‐blocks. The diameter variability due to the cutting angle was assessed by the cut slices from pre‐implant MEW‐cryo‐blocks, thereby comparing the cross‐sectional diameter with the original mesh‐fiber diameter. The diameter variability due to the cutting angle was assessed by the cut slices from pre‐implant MEW‐cryo‐blocks, thereby comparing the cross‐sectional diameter with the original mesh‐fiber diameter (Figure S6, Supporting Information). Aluminum foil‐wrapped slides were imaged under SEM (Nova NanoSEM, FEI, USA) following the same settings mentioned in section 5.2 to investigate fiber diameter in vivo assessing initial hydrolytic degradation and the pattern of neo‐collagen deposited following the implantation. Sample processing for SEM imaging followed similar steps described in section 5.3 except using cryosectioned tissue slices deposited on aluminum foil‐wrapped slides. After finishing the processing steps, samples were mounted over carbon tape and sputter‐coated with platinum particles for SEM imaging. Fiber diameter was measured using ImageJ for n = 5 tissue/mesh slices/group with a scale bar obtained from SEM imaging. The cross‐sectional areas of ten fibers in the mesh‐tissue border were measured and then equated with the circular cross‐sectional area (πr^2^; where r = radius of the corresponding fiber) to calculate the approximate diameter of the fibers. The complete set of data thus obtained was plotted in a frequency histogram and was averaged for statistical analyses.

In parallel, bare poly‐l‐Lysine slides were used to assess macrophage‐mediated foreign body response by immunofluorescence following the previously published protocols.^[^ ^15^, ^59^ ^]^ Briefly, immunofluorescence staining for total macrophages was with the F4/80 rat anti‐mouse pan macrophage antibody (Alex Fluor488 conjugated, 1 µg mL^−1^, eBioscience) diluted 1:200 and Rat IgG2a was used as a negative isotype control applied at the same concentration for 1 h at RT and was conjugated with Alex Fluor 488. Pro‐inflammatory M1 macrophages were detected with a CCR7 antibody (rabbit monoclonal, Abcam, Cat# Ab171870) diluted in 1:500 incubated for 1hr which was conjugated with Alex Fluor 647. Anti‐inflammatory M2 macrophages were assessed using a rat anti‐mouse CD206 (Alex Fluor647 conjugated, 0.5 mg mL^−1^, Bio legend) at 5 µg mL^−1^ diluted 1:200 for 1 h. For negative control, rabbit IgG, polyclonal, and Isotype control (Abcam, Cat# ab171870) was used at the same concentration and was conjugated with Alex Fluor 647. Finally, the secondary antibody, Donkey anti‐Rabbit IgG (Alex Flour 647, Abcam, Cat# ab65851), diluted 1:500, was used for primary CCR 7 antibody and Isotype control slides. Hoechst 33342 at 1:1000 dilution was used to stain nuclei. Samples were scanned using an Olympus VS 120 Slide scanner and images were assessed in OlyVIA software [v2.9]. All the images were set at the same respective thresholds for the three channels; blue for Nuclei, green for F4/80, yellow for CCR7 and CD206, and saved in .tiff formats for the quantitative assessment using a programmed macro in Image J software [v1.53f51]. Briefly, the regions of interest (ROIs) of the corresponding meshes were manually drawn by “Wait for user instruction command in macro” to specify the area in and around the mesh fibers including F4/80, CCR7, and CD206 positive immune cells. The total number of cells within a respective ROI were identified by the nuclei stain (Hoechst 33342). The multi‐color immunofluorescent images were processed in “Split Channel” operation to convert the respective binary images specific to the particular nuclei and immunostains. Next, optimal thresholding algorithm along with binary operations; “Despeckle”, “Remove outliers”, “Close” and “Analyse particles” were applied to the images. Finally, the “Summary” window was saved to obtain the count of total cells (nuclei stain) and other immune cells; F4/80^+^, CCR7^+,^ and CD206^+^ cells. The number of cells was normalised to respective ROIs. For sheep‐derived mesh‐tissue explants, pro‐inflammatory M1 and anti‐inflammatory M2 macrophages were detected by mouse anti‐sheep HLA‐DR and CD206 respectively, expression on PFA fixed OCT frozen blocks. The cryosectioned (5 µm) poly‐l‐Lysine slides were stained with mouse anti‐sheep HLA‐DR and CD 206. HLA‐DR and CD 206 were conjugated with Alexa‐Fluor‐488 donkey anti‐mouse and Alexa‐Fluor‐568 donkey anti‐mouse secondary antibodies in 30 min incubation at RT. Nuclei were stained with Hoechst 33258 for 3 min and the slides were mounted with a fluorescent mounting medium for imaging in Olympus VS 120 Slide scanner as mentioned above. Ex vivo mice tissues were collected and stored in RNA*later* (ThermoFisher) at 4 °C for 24 h and transferred to −80 °C for longer storage. Total RNA extraction, thereby, fluidigm 96.96 real‐time genes expression by qPCR was performed as per the published protocol. Briefly, the RNeasy mini kit (Qiagen) was used to extract total RNA following the manufacturer's protocol to prepare cDNA. Before qPCR, genes were amplified to increase the number of copies for detection. TaqMan PreAmp Master Mix (Life Technologies) was combined in the final reaction volume for final PCR workflow. Ct values were used to quantify relative gene expressions and fold change using 2^−ΔΔCT^ method. The genes were selected to specifically study key tissue repair and regeneration processes such as ECM formation, matrix regulation, angiogenesis, and FBR.

### Statistical Analysis

Statistical analysis was performed using Graph Pad Prism software version 7.0. Data were pre‐processsed for the normality assessments and outliers identifications of all data acquired in the different studies. Normality assessments were performed using the D'Agostino‐Pearson omnibus normality and the Shapiro‐Wilk normality tests. Data outliers were detected by “Identify Outliers”, a built‐in software program in Graph Pad Prism. Data are presented as mean ± SD. For in vitro MTS study, two‐way ANOVA with Tukey's post‐hoc analysis was used. One‐way ANOVA with Tukey's post‐hoc analysis was applied for mesh characterisation data and tensile loading data. The work done/functionality of MEW meshes was assessed by two‐way ANOVA with Sidak multiple comparisons test with median ± IQR (interquartile range) data presentation. In vivo data comparisons were performed by a group for each histologic time point and two‐way ANOVA with Tukey's post‐hoc analysis was applied, *p* < 0.05 was considered significant.

## Conflict of Interest

The authors declare no conflict of interest.

## Supporting information

Supporting Information

## Data Availability

The data that support the findings of this study are available on request from the corresponding author. The data are not publicly available due to privacy or ethical restrictions.
