# Supplementary material for: 3D Printed Mesh Geometry Modulates Immune Response and Interface Biology in Mouse and Sheep Model: Implications for Pelvic Floor Surgery
Source: Adv Sci (Weinh). 2024 Sep 19;12(11):2405004. doi: 10.1002/advs.202405004 (PMC11923936; doi:10.1002/advs.202405004)
Supplement: Supplementary file 1 — Supporting Information [file ADVS-12-2405004-s001.docx]

**3D Printed Mesh Geometry Modulates Immune Response and Interface Biology in Mouse and Sheep model: Implications for Pelvic Floor Surgery**

Kallyanashis Paul^1,2^, Saeedeh Darzi^1,2^, Cathal D O'Connell^3,4^, David M. Z. B. Hennes^1,2,5^, Anna Rosamilia^1,2,5^, Caroline E Gargett^1,2^, Jerome A Werkmeister^1,2^ and Shayanti Mukherjee^1,2,*^

*^1^The Ritchie Centre, Hudson Institute of Medical Research, Clayton, Australia 3168;*

*^2^Department of Obstetrics and Gynaecology, Monash University, Clayton Australia 3168;*

*^3^Biofab3D@ACMD, St Vincent's Hospital, Melbourne, VIC, Australia*

*^4^Discipline of Electrical and Biomedical Engineering, School of Engineering, RMIT University, Melbourne, VIC, Australia 3000*

*^5^Pelvic Floor Disorders Unit, Monash Health, Clayton, VIC, Australia 3168*

*****Corresponding Author**

A/Prof. Shayanti Mukherjee

Al and Val Rosenstrauss Fellow

Principal Research Scientist & Lab Head

Translational Tissue Engineering Lab,

The Ritchie Centre

Hudson Institute of Medical Research

Translation Research Facility

Level 5 27-31 Wright Street, Clayton, Victoria, 3168 Australia

Phone: +61 3 8572 2795

Email: [shayanti.mukherjee@monash.edu](mailto:shayanti.mukherjee@monash.edu); [shayanti.mukherjee@hudson.org.au](mailto:shayanti.mukherjee@hudson.org.au)

9.0 Supplementary Data

### Table S1: Type and nomenclature of MEW meshes

| **No** | **Mesh description of vertical stacking** | **Mesh Name** | |
| --- | --- | --- | --- |
| 1 | 90^O^ 1 mm space 50 layers; 1 pore mesh | | 90^O^1P |
| 2 | 45^O^ 1 mm space 50 layers; 1 Pore mesh | | 45^O^1P |
| 3 | 22.5^O^ 1 mm space 50 layers; 1 Pore mesh | 22.5^O^1P | |
| 4 | 90^O^ 0.5 mm space 50 layers; 1 Pore mesh | 90^O^0.5P | |
| 5 | 45^O^ 0.5 mm space 50 layers; 1 Pore mesh | 45^O^0.5P | |
| 6 | 22.5^O^ 0.5 mm space 50 layers; 1 Pore mesh | 22.5^O^0.5P | |
| 7 | 90^O^ 1mm space 25 layers + 0.5mm space 25 layers; hierarchical mesh | 90^O^2P | |
| 8 | 45^O^ 1 mm space 25 layers + 0.5mm space 25 layers; hierarchical mesh | 45^O^2P | |
| 9 | 22.5^O^ 1 mm space 25 layers + 0.5mm space 25 layers; hierarchical mesh | 22.5^O^2P | |
| 10 | Non-porous sheet construct; No Pore mesh | No Pore | |


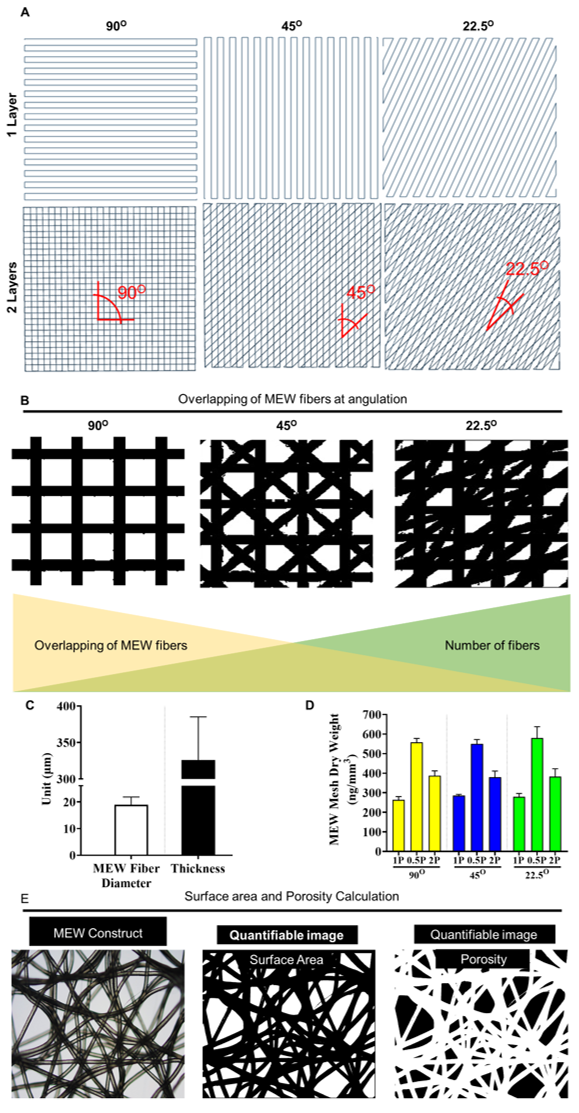


**Figure S1: Schematic of layer-by-layer addition in MEW process** showing (A) the angle between the successive layers depicted in the first two layers, (B) overlapping of MEW fibers at angulation, MEW characterisations showing (C) MEW fiber diameter and thickness of 50 layers, (D) normalised MEW weight and (E) quantifiable image of bioactive surface area and porosity calculation.


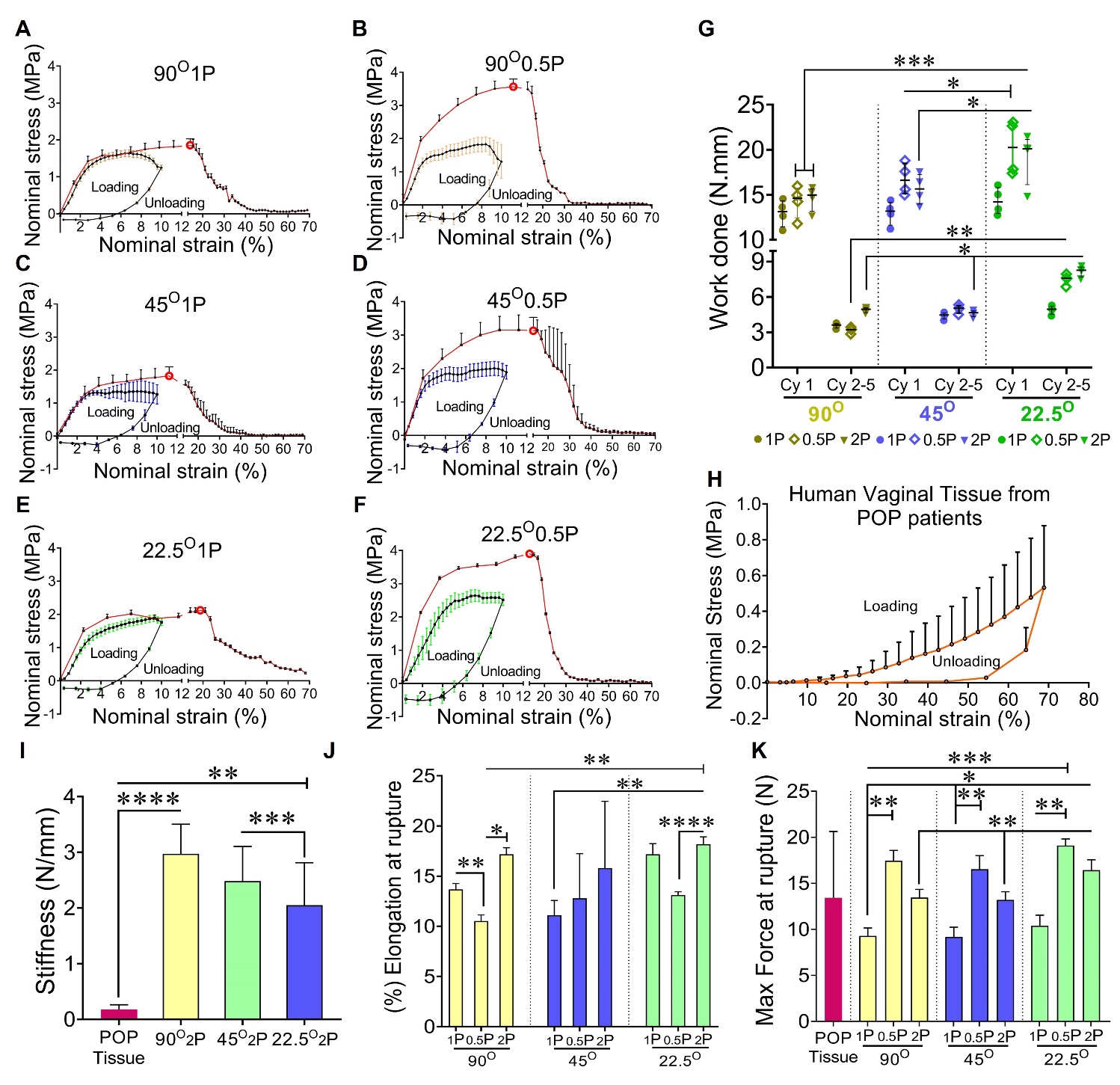


**Figure S2: Mechanical characterisations of MEW meshes under tensile loading** showing (A-F) stress-strain curve under cyclic loads at 10% strain (black solid lines) and breaking loads at 70% strain (red solid lines); pink bordered black dots are yield points and red bordered white dots are the breaking/tearing points, (G) work-done under cyclic loading, (H) nominal stress-strain curve of prolapsed tissues collected from human patients with severe POP, (I) stiffness of prolapsed tissues and hierarchical MEW meshes, (J) percentage of elongation and (K) maximum force at rupture. Data are mean ± SD, n= 5 samples/group. Statistical analysis is one-way ANOVA with Tukey's multiple comparisons test, (*P<0.05; **P<0.0035; ***P<0.0003; ****P<0.0001). For toughness assessment, data are median ± interquartile range (IQR). Statistical analysis is two-way ANOVA with Sidak multiple comparisons test, (*P<0.05; **P<0.009; ***P<0.0004; ****P<0.0001).

#### Table S2: Tensile properties of MEW meshes

| **Angle** | **Inter-fiber Space** | **Stiffness (N/mm)** | **Elastic Modulus (MPa)** |
| --- | --- | --- | --- |
| **90^O^** | **1P** | 1.24 ± 0.3 | 16.89 ± 2.4 |
|  | **0.5P** | 3.25 ± 0.4 | 19.53 ± 1.56 |
|  | **2P** | 2.9 ± 0.5 | 15.88 ± 1.67 |
| **45^O^** | **1P** | 1.15 ± 0.4 | 11.31 ± 1.45 |
|  | **0.5P** | 2.36 ± 0.6 | 27.8 ± 2.68 |
|  | **2P** | 2.4 ± 0.6 | 15.44 ± 1.79 |
| **22.5^O^** | **1P** | 1.21 ± 0.4 | 11.7 ± 1.56 |
|  | **0.5P** | 2.28 ± 0.6 | 35.45 ± 3.35 |
|  | **2P** | 2.04 ± 0.7 | 16.21 ± 1.78 |

#### Data are mean ± SD for n=5/group

#### Table S3: Sample description for the isolation of vaginal fibroblasts

| Samples | Non-POP Patients | |
| --- | --- | --- |
| No | Age | POP Stage |
| 1 | 86 | 0 |
| 2 | 84 | 0 |
| 3 | 64 | 0 |
| 4 | 52 | 0 |
| 5 | 52 | 0 |
| Average | 67.6 |  |
| SD | 16.6 |  |


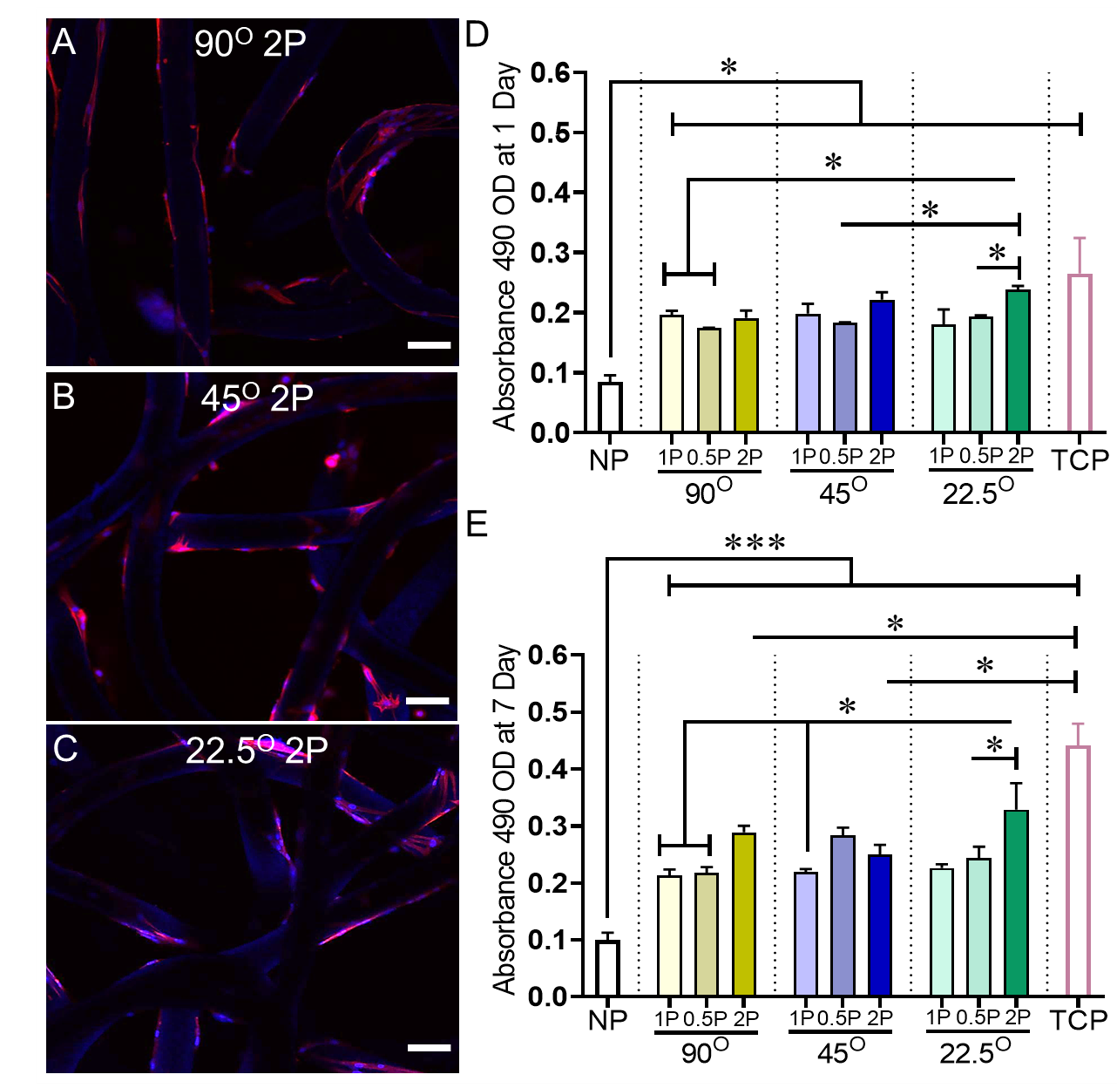


**Figure S3: *In vitro* cell proliferation assay** showing (A-C) cytosketal expression (actin filaments; red color) of vaginal fibroblasts at day 14, (D-E) proliferation of vaginal fibroblasts by MTS assay at day 1 and 7. NP represents “no pore” mesh, and TCP represents “tissue culture plate”. Data are mean ± SD. Statistical analysis is two-way ANOVA with Tukey's multiple comparisons test, (*P<0.05; ***P<0.004).

| **Table S4: Distribution of 46 mice across the sample groups** | | | |
| --- | --- | --- | --- |
|  |  | **Two Timepoints; 1 & 6 Wks** | |
| **Angle** | **Inter-fiber Space** | **N Numbers (Mice)** | **N numbers Mesh** |
| **90^O^** | **1P** | 4 | 8 |
|  | **0.5P** | 4 | 8 |
|  | **2P** | 6 | 12 |
| **45^O^** | **1P** | 4 | 8 |
|  | **0.5P** | 4 | 8 |
|  | **2P** | 6 | 12 |
| **22.5^O^** | **1P** | 4 | 8 |
|  | **0.5P** | 4 | 8 |
|  | **2P** | 6 | 12 |
| **NP (Neg Control)** | **Not applicable** | 4 | 8 |


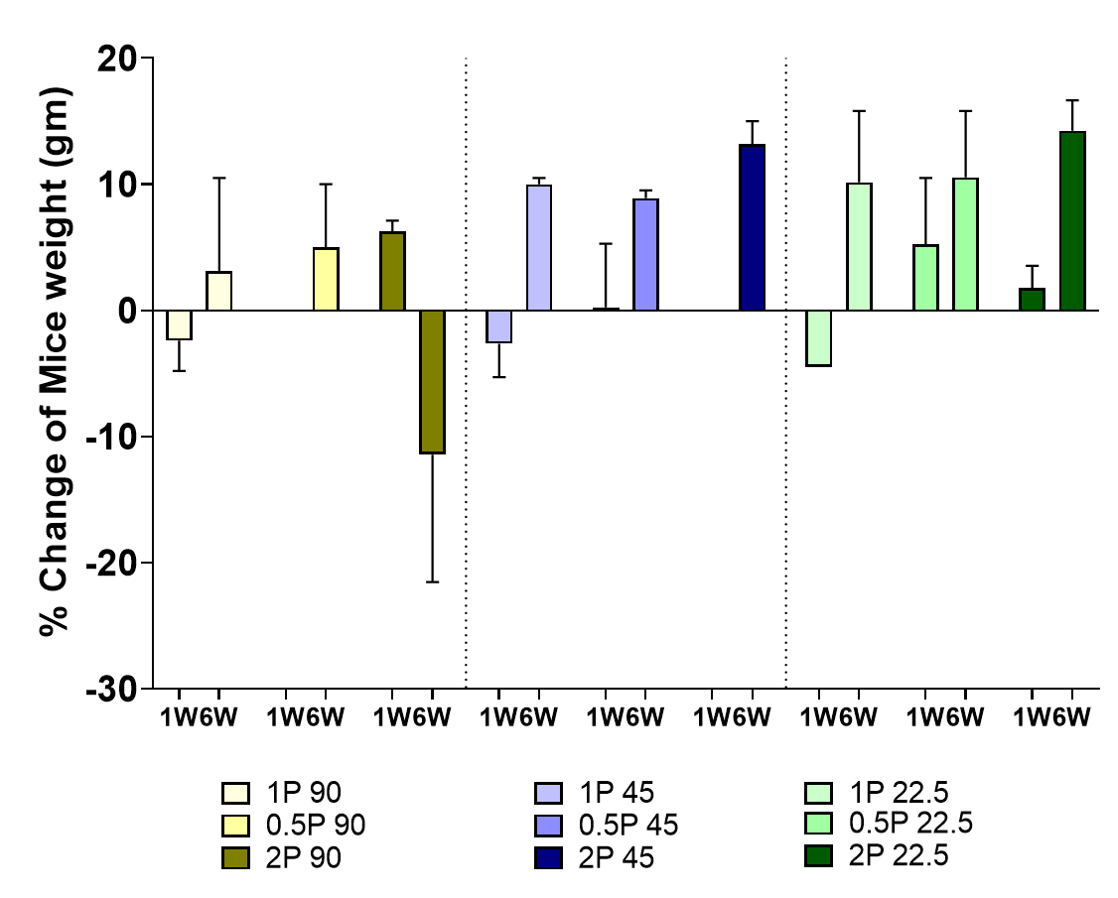


**Figure S4: Change of mice weight at histologic endpoints at 1 and 6 Wk.** Data are mean ± SEM, n=6 meshes/group (2P) and n=4 meshes/group (1P, 0.5P).


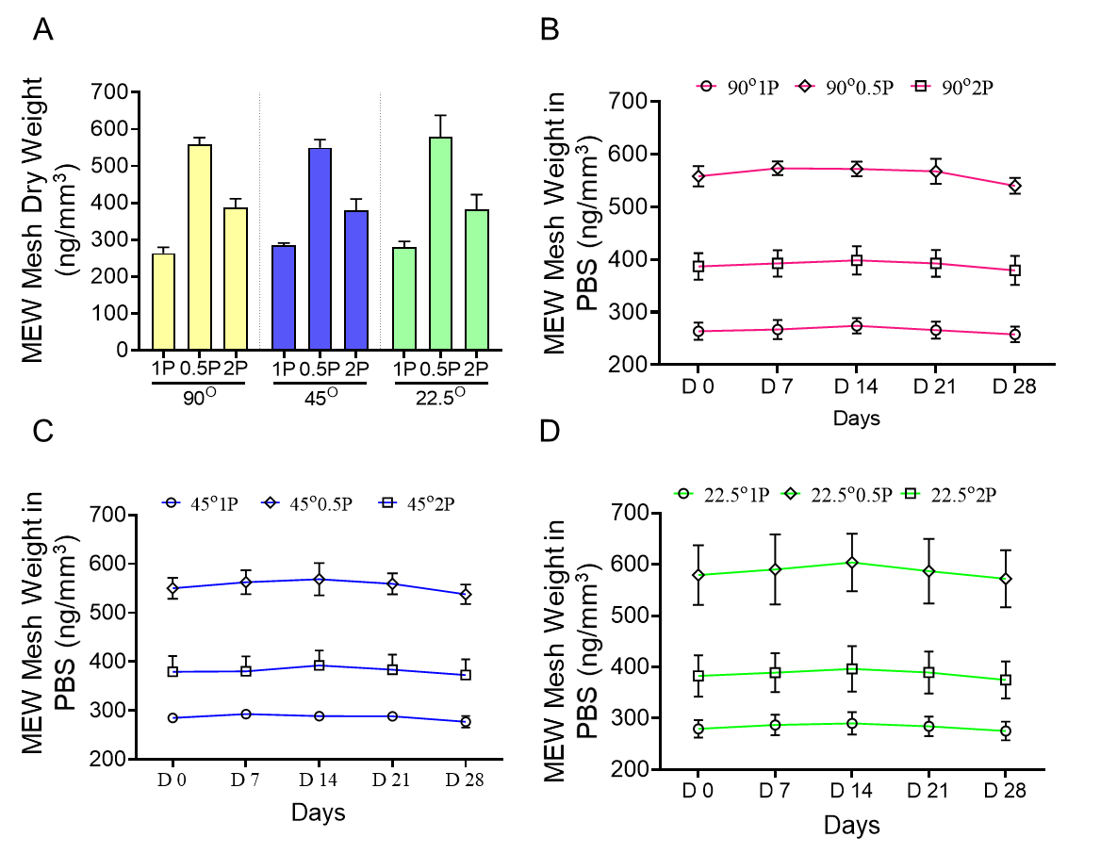


**Figure S5: In vitro degradation of MEW meshes** showing (A) dry weights, (B-D) weights after soaking in PBS at day 7, 14, 21 and 28. Weights are in nanogram (ng) normalised to volume (mm^3^). Data are mean ± SD, n=5 meshes/group.


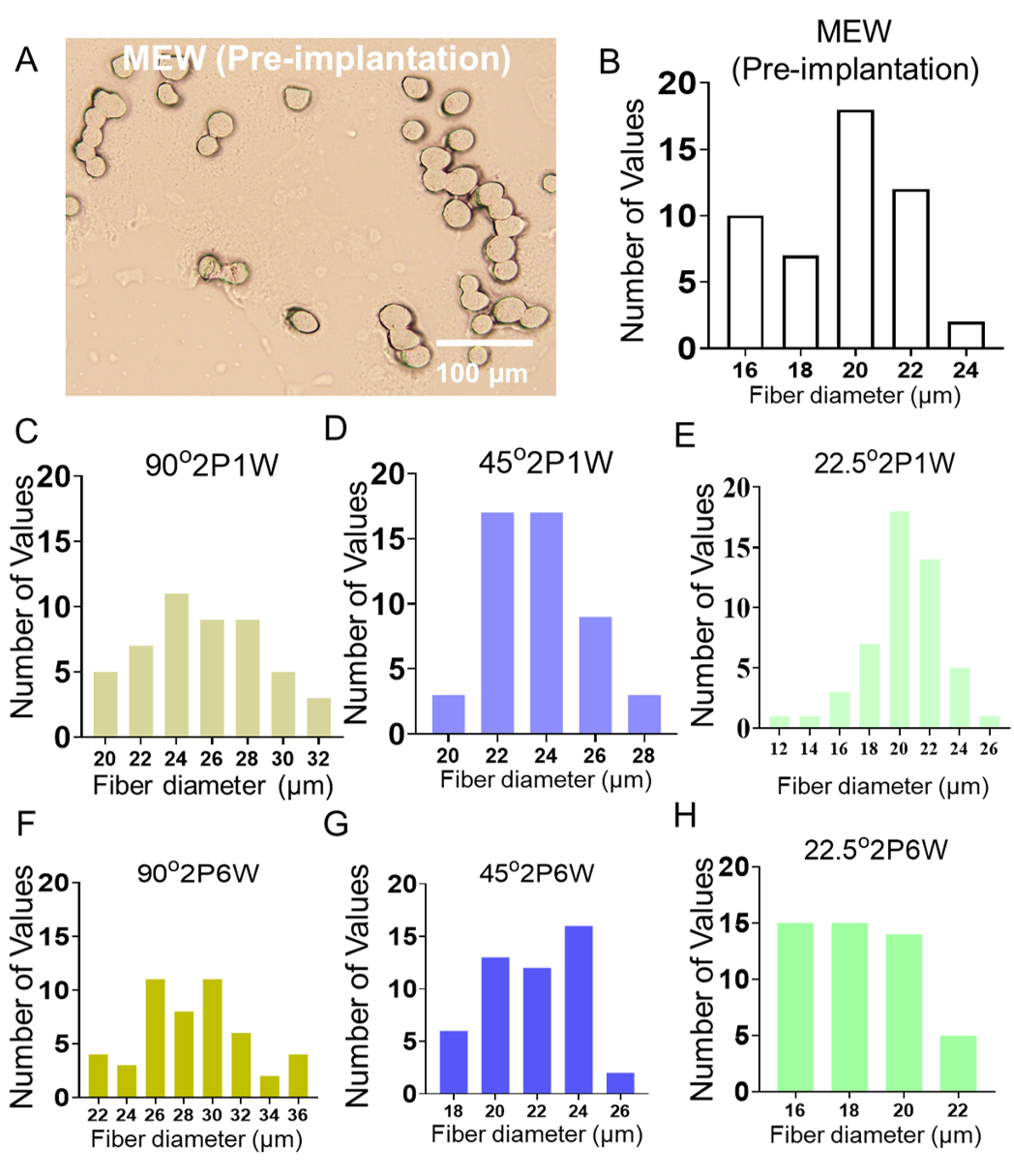


**Figure S6: Histogram of OCT embedded MEW fiber diameter** showing frequency distribution of (A, B) MEW fibers after cross-sectional cutting, (C-E) MEW fibers of 2P meshes after 1Wk ex vivo and (F-H) MEW fibers of 2P meshes after 6Wk ex vivo.


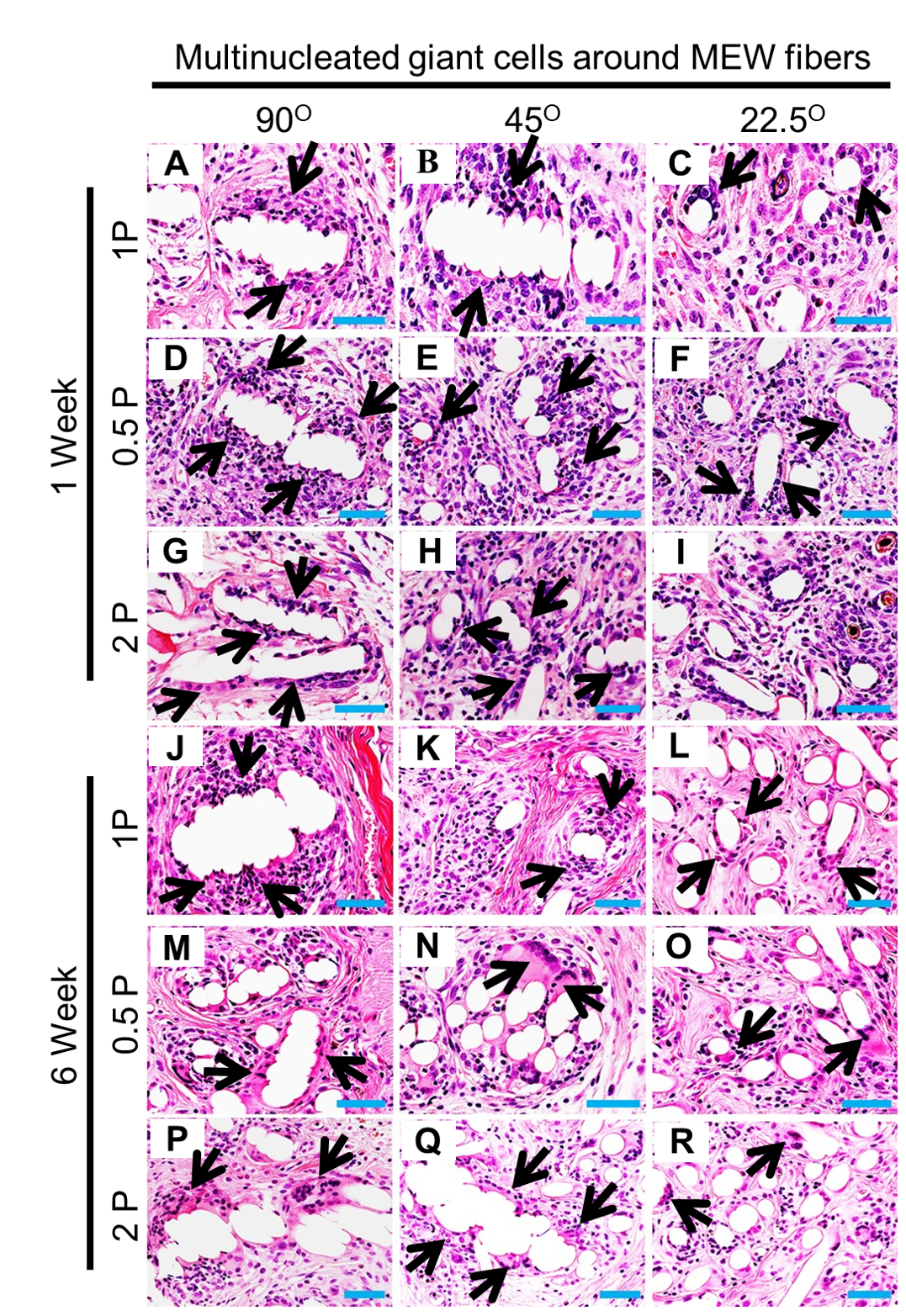


**Figure S7: The presence of multinucleated giant cells due to angulation after 1 and 6 Wk** showing the spreading and qualitative distribution of multinucleated giant cells (A-R). Black arrows represent the giant cells across different meshes. Blue scale bars are 50 µm.


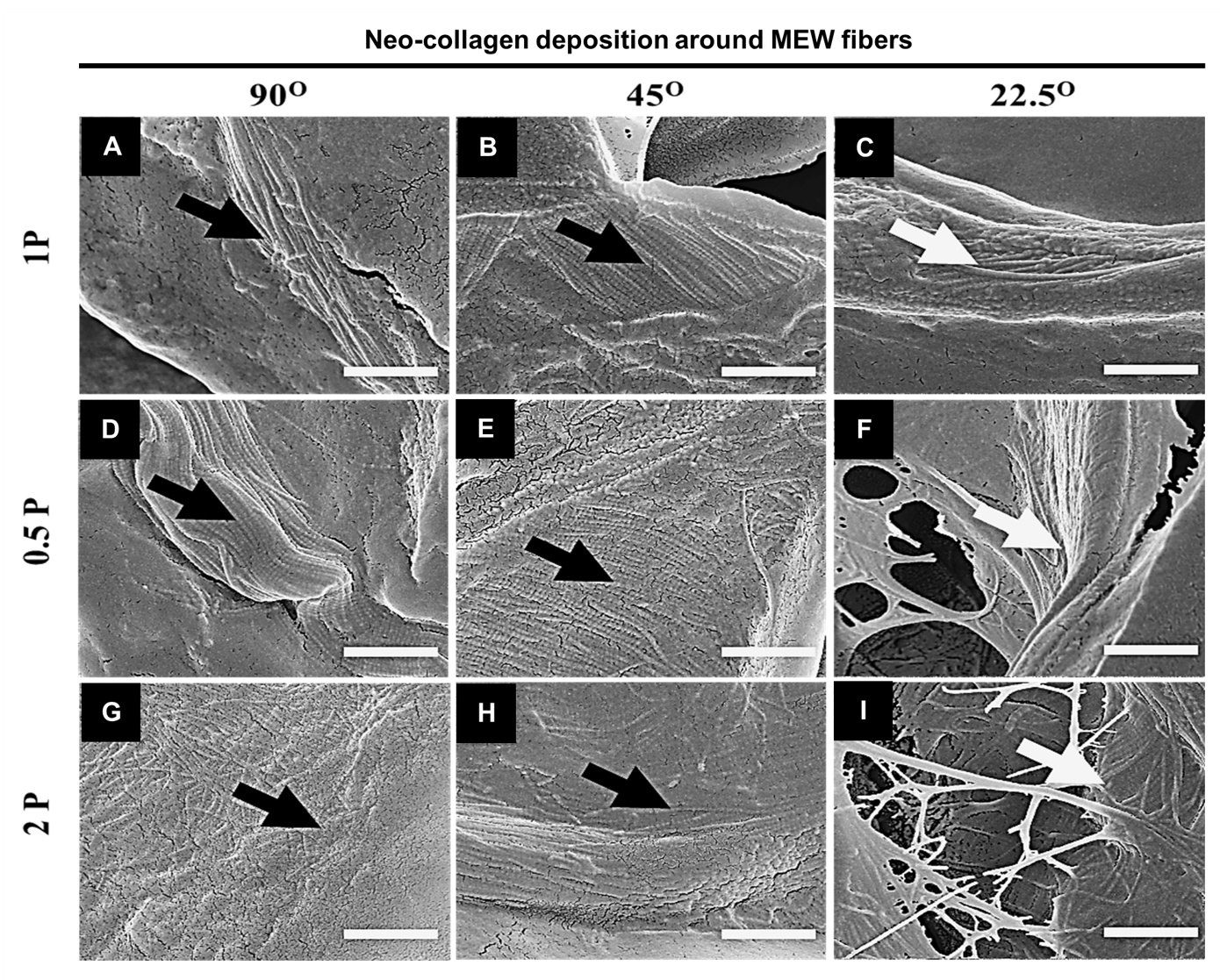


**Figure S8: Collagen deposition due to the effect of angulation after 6 Wk** showing the pattern of neo-collagen deposition (A-I) by black arrows for 90^O^ and 45^O^ meshes and by white arrows for 22.5^O^ meshes. White scale bars are 1 µm.


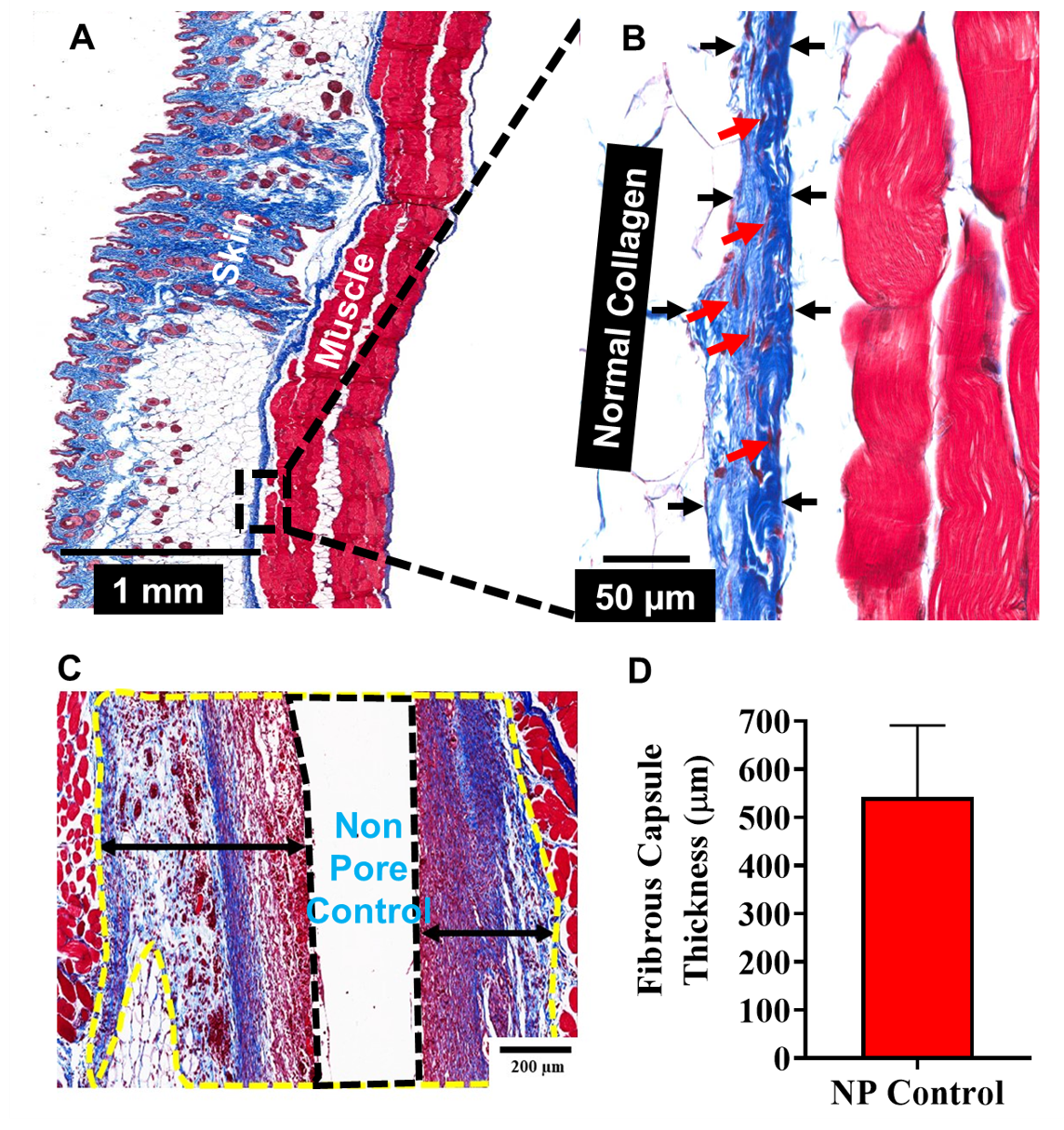


**Figure S9: (A) Negative control of Skin explant excluding meshes** showing the extent of normal collagen (black arrows) at the skin-muscle interface and mono nuclear skin fibroblast (red arrows) on the magnified image (B). Positive control of fibrous capsule formation with thickness (black arrows) in non-porous mesh-tissue explants is represented in (C-D). Data are mean ± SD.


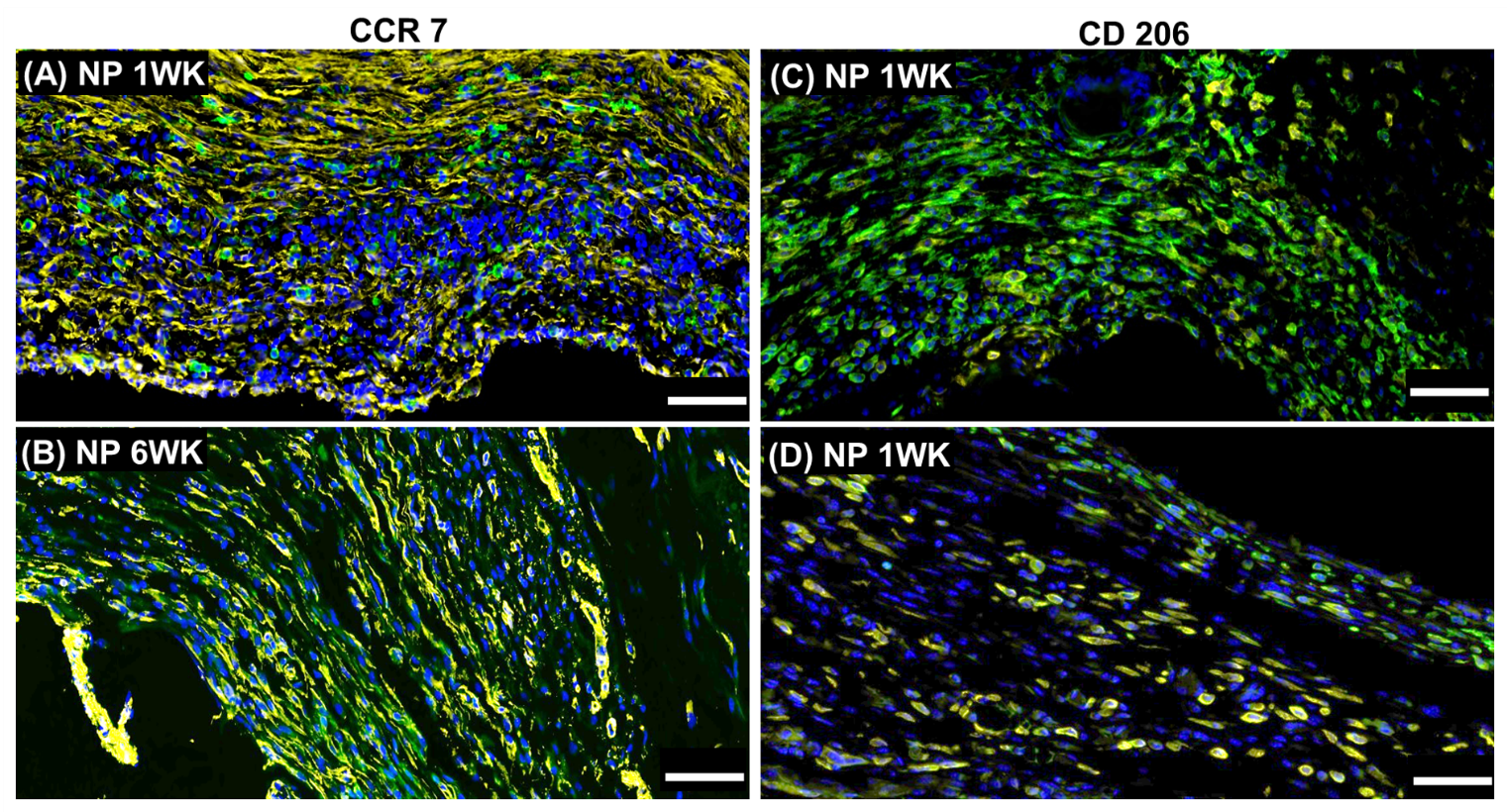


**Figure S10: Macrophage-associated foreign body response in non-porous (NP) meshes after 1 and 6 Wk,** showing (A-B) pro-inflammatory M1 macrophages (yellow) and (C-D) anti-inflammatory M2 macrophages (yellow). Scale bars are 50 µm. Cell nuclei in blue and pan macrophage; F4/80+ cells in green.


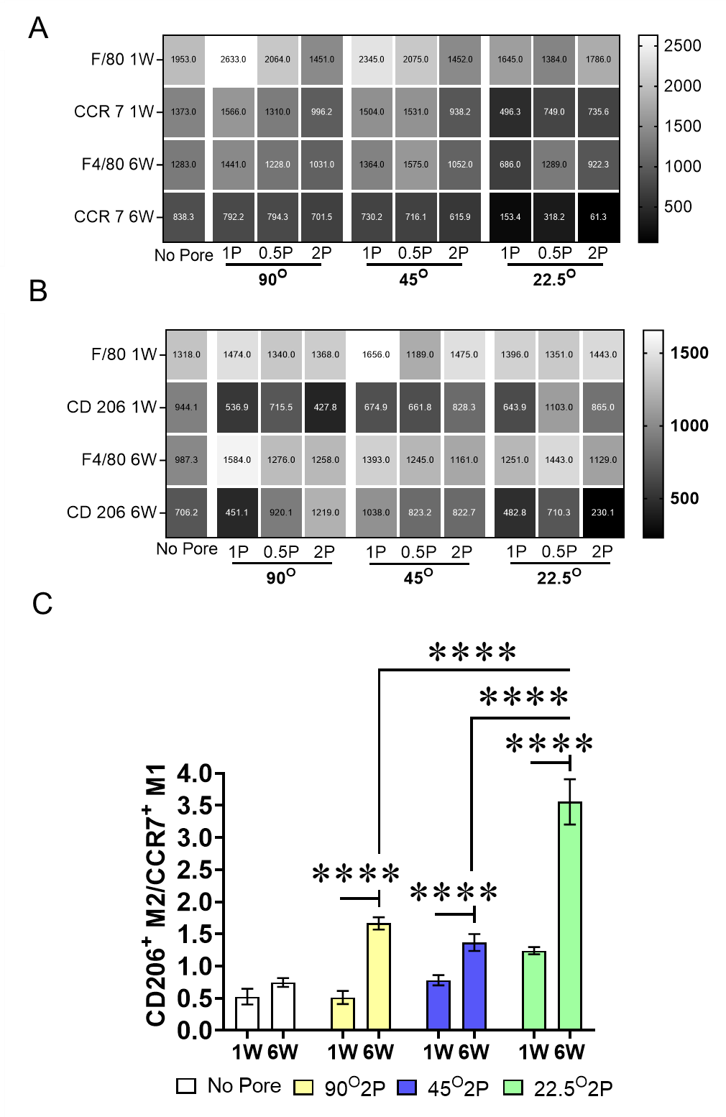


**Figure S11: Summary of macrophage-associated foreign body response after 1 and 6 Wk showing** heatmap (A-B) of pro-inflammatory CCR7^+^ (M1) and anti-inflammatory CD206^+^ (M2) macrophages. The percentage population of M1 and M2 macrophages due to the angulation is summarised in (C-D). Data are mean ± SD, n= 6/group.


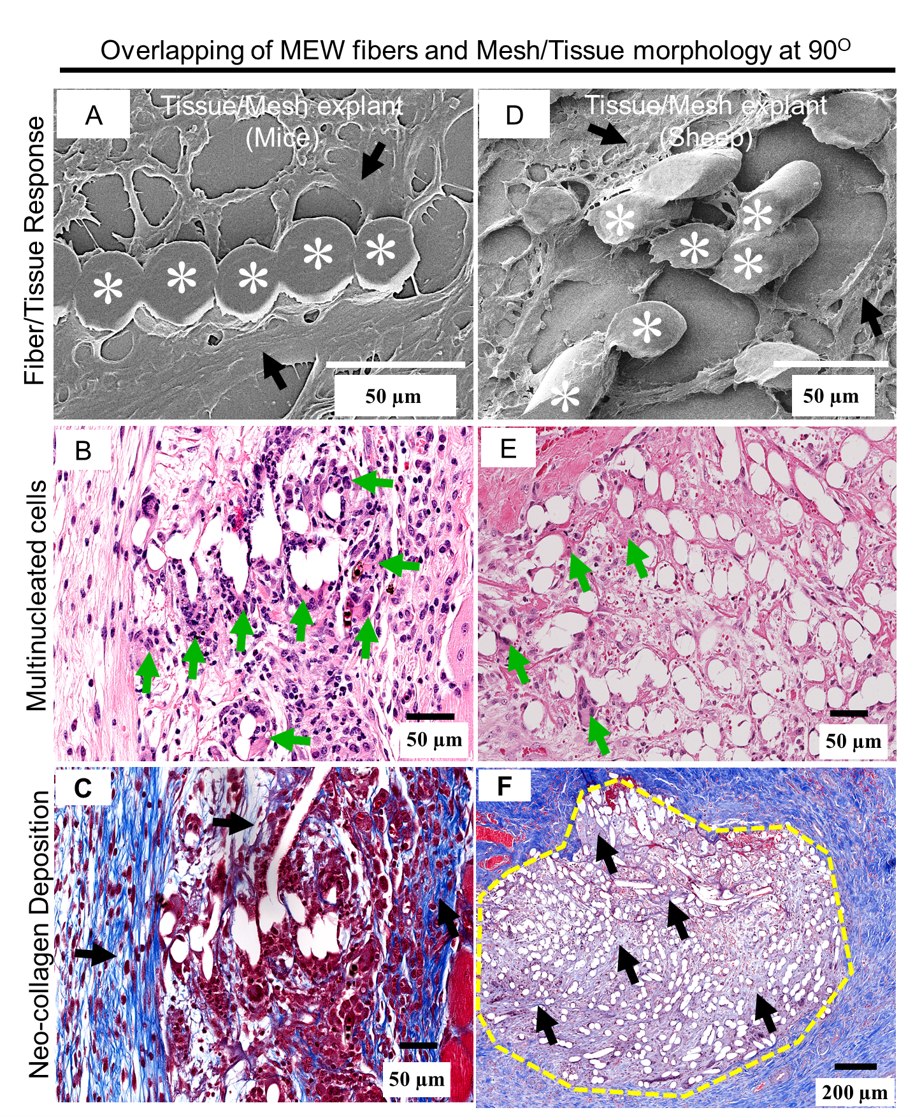


**Figure S12: Tissue response in mouse (A-C) and sheep model** **(D-F)** showing (A, D) SEM images for the similar collagen deposition (black arrows) due to the overlapping of 90^O^ mesh fibers (white asterisks) at 1 Wk tissue explant. (B, E) multinucleated giant cells (green arrows) and (C, F) showing neo-collagen deposition associated (black arrows) with the same 90^O^ fibers stacking.
